# Universal insertion of molecules in ionic compounds under pressure

**DOI:** 10.1093/nsr/nwae016

**Published:** 2024-01-11

**Authors:** Feng Peng, Yanming Ma, Chris J Pickard, Hanyu Liu, Maosheng Miao

**Affiliations:** College of Physics and Electronic Information, Luoyang Normal University, Luoyang 471022, China; Department of Chemistry and Biochemistry, California State University Northridge, Northridge 91330, USA; State Key Laboratory of Superhard Materials & Key Laboratory of Material Simulation Methods and Software of Ministry of Education, College of Physics, Jilin University, Changchun 130012, China; International Center of Future Science, Jilin University, Changchun 130012, China; Department of Materials Science & Metallurgy, University of Cambridge, Cambridge CB3 0FS, UK; Advanced Institute for Materials Research, Tohoku University, Sendai 980-8577, Japan; State Key Laboratory of Superhard Materials & Key Laboratory of Material Simulation Methods and Software of Ministry of Education, College of Physics, Jilin University, Changchun 130012, China; International Center of Future Science, Jilin University, Changchun 130012, China; Department of Chemistry and Biochemistry, California State University Northridge, Northridge 91330, USA; Department of Earth Science, University of California Santa Barbara, Santa Barbara 93106, USA

**Keywords:** molecule-solid hybrid materials, high-pressure, crystal structure prediction, density functional theory, planet interior

## Abstract

Using first-principles calculations and crystal structure search methods, we found that many covalently bonded molecules such as H_2_, N_2_, CO_2_, NH_3_, H_2_O and CH_4_ may react with NaCl, a prototype ionic solid, and form stable compounds under pressure while retaining their molecular structure. These molecules, despite whether they are homonuclear or heteronuclear, polar or non-polar, small or large, do not show strong chemical interactions with surrounding Na and Cl ions. In contrast, the most stable molecule among all examples, N_2_, is found to transform into cyclo-N_5_^−^ anions while reacting with NaCl under high pressures. It provides a new route to synthesize pentazolates, which are promising green energy materials with high energy density. Our work demonstrates a unique and universal hybridization propensity of covalently bonded molecules and solid compounds under pressure. This surprising miscibility suggests possible mixing regions between the molecular and rock layers in the interiors of large planets.

## Teaser

Calculations show a universal reaction under high pressure in which various molecules, despite their size and polarity, can insert into ionic crystals and form stable compounds without strong chemical interactions.

## INTRODUCTION

Chemical substances are generally divided into two large categories: molecules and solid-state compounds. They distinctly differ in structures, bonding features, and properties and have been used in different areas. Molecules are generally formed from nonmetals and are held together by covalent bonds, whereas solid-state compounds often comprise metals or metalloids and can be characterized by ionic or metallic bonding. Hybrid materials, which consist of inorganic components and small molecules, have gained intensive attention owing to their unique chemical structure, physical properties, and potential applications in optics, electronics, mechanics, catalysis, and sensors as well as biomedical devices [1–5]. However, these unique characteristics also impose challenges on material synthesis, characterization, as well as the fundamental understanding of their chemical behavior.

The formation of most hybrid materials usually is caused by strong chemical interactions between inorganic species, such as ions and atoms, and small organic molecules. For instance, well-known hydrate compounds can be considered as a class of hybrids in which water molecules (H_2_O) are chemically integrated into inorganic crystal structures. Notably, CuSO_4_ forms hydrates of the form CuSO_4_·*n*H_2_O where *n* can range from 1 to 7 [6]. In these compounds, the lone-pair electrons in H_2_O form coordinate bonds with the *d* orbitals of transition metal hosts. NaCl, a classic ionic solid, can also form hydrates, especially under elevated pressure conditions. This phenomenon is driven by the screening of electrostatic potentials by highly polar molecules such as H_2_O. This interaction mirrors the solvation of NaCl in polar solvents, whereby water molecules surround and isolate individual sodium and chloride ions.

One of the more recent and notable examples of hybrid materials is organic-inorganic hybrid perovskites. These materials have gained significant attention due to their promising efficiency as photovoltaic materials. The formation of these perovskites is largely attributed to the strong ionic interactions between the negatively charged inorganic anions and the positively charged organic cations. This hybrid structure offers a promising route toward the design of next-generation energy materials [7].

In the current investigation, we aim to explore a distinctive new category of hybrid materials whose formation is not facilitated by strong chemical interactions between molecules and the encompassing inorganic species. Our study was inspired by recent research into the unique chemistry that emerges under high pressure. Under these conditions, the chemical properties of elements and the strengths of the homonuclear and heteronuclear bonds can change drastically, leading to the formation of many atypical compounds with non-intuitive compositions and structures, like NaCl_3_, Fe_3_Xe, and CsF_3_ [8–10].

Moreover, many recent theoretical and experimental studies have revealed that helium (He) can react with various ionic crystals to form stable ternary compounds under high pressure. In these compounds, He, despite being a noble gas, is integrated into the crystal structure without forming localized bonds with neighboring atoms. In this context, several pressure-stabilized He compounds have been predicted or synthesized, such as MgF_2_He, FeO_2_He, and Na_2_He [11–13]. These observations signal an unprecedented capacity of He to interact with other substances under high-pressure conditions, despite its known chemical inertness.

In this study, we aim to expand upon this concept of helium insertion with a view to investigating the potential for inserting small molecules, e.g. H_2_, N_2_, CO_2_, NH_3_, H_2_O, and CH_4_*et al*., into ionic compounds under pressure. The examples are carefully chosen to represent homonuclear and heteronuclear molecules, non-polar and polar molecules, and molecules with different sizes. Unlike helium, which is highly resistant to forming chemical bonds, these small molecules generally have a much higher chemical reactivity. However, our comprehensive crystal structure search studies reveal a compelling general chemical trend under high-pressure conditions. Despite their higher chemical activity compared to helium, most of these small molecules surprisingly retain their integrity when inserted into prototypical ionic compounds, such as NaCl. This leads to the formation of thermodynamically stable hybrid compounds, opening up exciting new possibilities in exploring unconventional hybrid materials.

An immediate application of our study is the understanding of the composition and structure of planets’ interiors. All large planets consist of both covalently bonded molecules and solid-state minerals, segregated into different layers with large dispersive regions. Mars and Venus consist of three layers: a rocky (iron-nickel/sulfide) core in the center, a silicate mantle in the middle and an outer composed mainly of gaseous carbon dioxide (CO_2_) and nitrogen (N_2_). The interiors of Uranus and Neptune are mainly composed of a rocky (silicate/iron-nickel) core in the center and icy mantle (water, ammonia, and methane, along with traces of other hydrocarbons, but not necessarily for these molecules). There are abundant ionic compounds and small molecules in the boundary of mantle-outer layer (Mars and Venus) and core-mantle (Uranus and Neptune) boundaries. Our investigations on structures and physical properties of salt-SM hybrid compounds will provide key information to the understanding of the interior structure and dynamics of these planets. Especially, minerals and SM both exist in a variety of ocean exoplanets’ interiors, namely salty high-pressure ice (NaCl-H_2_O) [14,15].

To this end, we performed extensive structure searches to examine the possibility of forming hybrid salt-SM materials under high pressure [16,17]. Our simulations uncovered a variety of stable NaCl-SM compounds with various compositions at a wide pressure range of up to 200 GPa. Among all predicted structures, NaCl(H_2_)_4_ and NaCl(N_2_)_5_ compounds could be stable at pressures of 38 and 36 GPa, respectively. Our current findings not only establish a new family of hybrid salt-SM compounds for further design and discovery of intriguing materials, especially for the combination of inorganic compounds and small organic molecules under high pressure, but also provide crucial implications for the understanding of the interior of exoplanets. In addition, NaCl is often employed as a pressure-transmitting medium and thermal insulator in diamond anvil cell experiments due to its high compressibility, low strength, and limited chemical reactivity as well as its general ease of use. The present results also provide useful suggestions on the effectivity and the chemical limit of a solid NaCl pressure-transmitting medium.

## RESULTS AND DISCUSSION

**Figure 1.**
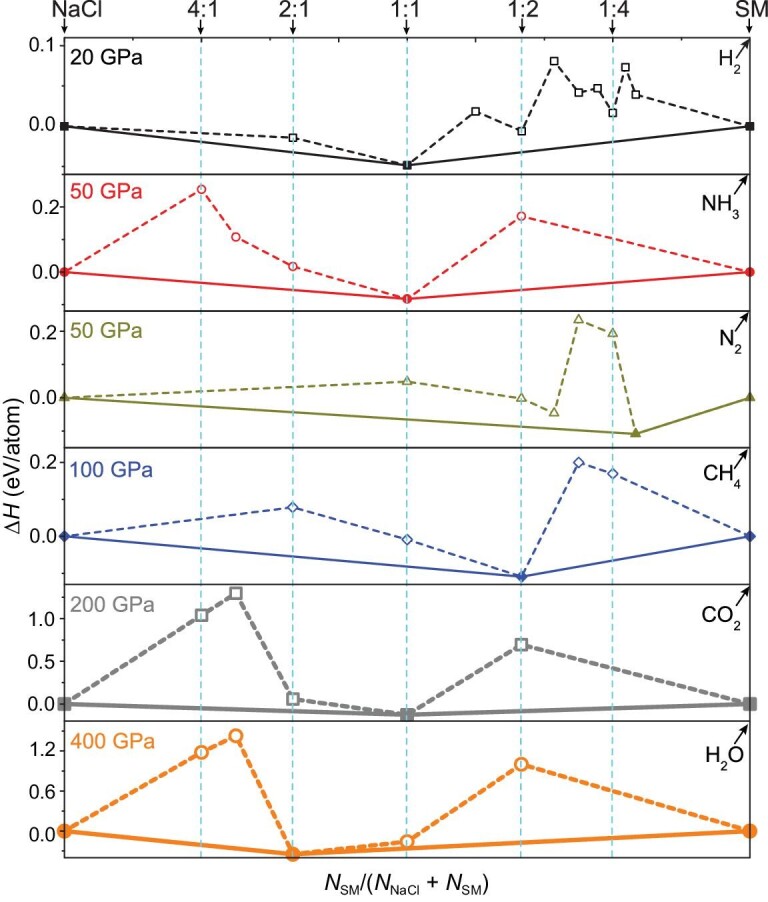
Phase stabilities of various NaCl-SM hybrid compounds. Enthalpies of formation of NaCl-SM (SM = H_2_, N_2_, CO_2_, NH_3_, H_2_O, CH_4_) under several pressures. Dotted lines connect the data points, and solid lines denote the convex hull.

First, we conducted an extensive exploration on the high-pressure phase diagrams of NaCl-SM (SM = H_2_, N_2_, CO_2_, NH_3_, H_2_O, CH_4_) hybrid compounds at high pressures by performing swarm-intelligence based CALYPSO [18,19] structure searches. The thermodynamic stability of NaCl-SM hybrid compounds is evaluated from their formation enthalpies relative to the dissociation products of NaCl + SM. In principle, NaCl-SMs are ternary compounds and may have a great amount of different possible decomposition products. However, all structure searches show that there is no sign of SM dissociation at the pressures considered in this study, which allows us to treat SM as a single reaction unit. On the other hand, NaCl has the most stable stoichiometry among all metal halides at ambient conditions and the high-pressure range used in this study [20,21]. Therefore, we reduce the stability evaluation to a pseudo-binary reaction of *m*NaCl + *n*SM → *m*NaCl (SM)*_n_*. For NaCl, the known body-centered cubic and face-centered cubic structures are considered in their corresponding stable pressure ranges. The phases of *P*6_3_/*mc, ε*-N, *I*-42*d*, P2_1_2_1_2, *Pbcm* and *Pnma* are considered for H_2_, N_2_, CO_2_, NH_3_, H_2_O, and CH_4_ in their corresponding pressure, respectively. As a consequence, our simulations identified a series of hitherto unknown NaCl-SM (SM = H_2_, N_2_, CO_2_, NH_3_, H_2_O, CH_4_) hybrid compounds under high pressure, as shown in Fig. 1. In this work, we take hybrid NaCl(H_2_)_4_ compound as an example, since this structure is predicted to become thermodynamically stable at sub-megabar pressures of 38 GPa.

As shown in Fig. 1, at 20 GPa, NaClH_2_ is the only stable composition, but at a higher pressure of 100 GPa, both NaClH_2_ and NaCl(H_2_)_4_ (containing four H_2_ molecules) (Fig. S1) become stable. As the pressure increases from 20 to 100 GPa, the formation enthalpy of NaClH_2_ increases from −49 meV/atom to −192 meV/atom. These results indicate that pressure could significantly promote the formation of stable salt-H_2_ compounds with a higher H_2_ content. Besides, we also attempted to study other hybrid salt-H_2_ stabilities under high pressure, such as KI-H_2_, RbI-H_2_ and RbBr-H_2_ (Fig. S1). The stable pressure range of the hybrid salt-H_2_ compounds are summarized in Fig. S1d. It is remarkable that KI forms a hybrid compound with H_2_ at the lowest pressure of 1 GPa, and therefore the possibility of future experimentation is greatly stimulated. It is noted that the NaClH*_x_* compounds have been recently synthesized [22], in which our predicted NaClH_2_ with *P*6_3_/*mmc* symmetry [23] agrees well with the structure observed in the experiment (Table S2 and Fig. S13). This greatly encourages us to study other NaCl-SM hybrid compounds, although our predicted NaCl(H_2_)_4_ with *Pm* symmetry was not observed. We found that the hybrid salt-H_2_ compounds become less stable with increasing cation radius from NaCl to CsCl. In contrast to the trend of the cation, the increased size of the anion increases the stability of the hybrid salt-H_2_ compounds. These results indicate that the difference in the sizes of the cation and the anion is a determining factor in the formation of stable hybrid compounds with H_2_ molecules, which naturally explains the reason for the ultra-low-pressure threshold of KI-H_2_ (Fig. S1c). This offers an unexpected prospect for the storage of hydrogen in the salt, and other similar ionic compounds.

The above calculations fully include the effects of van der Waals (vdW) interactions and zero-point energies (ZPE), which may play critical roles in determining the stability of these predicted hydrogen-rich compounds. After considering these effects, the formation energies of the hybrid salt-H_2_ compounds remain almost unchanged, while transition pressures of these compounds are slightly different from those without considering these effects. For example, the phase transition pressure for the *Pc* to *P*6_3_/*mmc* structures is 14.6 GPa as compared to the static lattice PBE result of 16.5 GPa for NaClH_2_ (Fig. S27), and the formation pressures of NaClH_2_ are 15.3 and 17.4 GPa with and without vdW interactions, respectively. Once the ZPE is included, however, the formation pressure for NaClH_2_ becomes 19.7 GPa.

**Figure 2.**
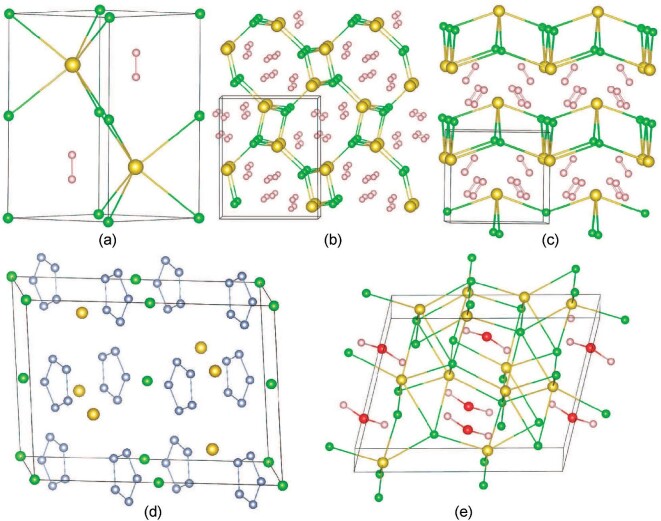
Structures of salt-SM hybrid compounds. Three different ways of inserting SM molecules inside the lattices of the NaCl: at atomic sites in NaClH_2_ with *P*6_3_/*mmc* symmetry at 20 GPa (a); inside tubes in NaI(H_2_)_2_ with *P*2_1_2_1_2_1_ symmetry at 50 GPa (b); and between layers in NaBr(H_2_)_2_ with *Pmmn* symmetry at 30 GPa (c). (d) NaClN_10_ with *C*2/*c* symmetry at 50 GPa; (e) (NaCl)_2_H_2_O with *C*2/*c* symmetry at 300 GPa. The red, pink, grey, gold and green spheres represent O, H, N, Na and Cl atoms, respectively.

Our structure searches reveal unique, and in many cases surprisingly simple, structural features for the salt-SM hybrid compounds. Depending on the concentration of H_2_, our predicted structures represent three different ways of inserting H_2_ molecules into the lattices of the salt: at atomic sites (Fig. 2a, NaClH_2_ with *P*6_3_/*mmc* symmetry), inside tubes (Fig. 2b, NaI(H_2_)_2_ with *P*2_1_2_1_2_1_ symmetry), and between layers (Fig. 2c, NaBr(H_2_)_2_ with *Pmmn* symmetry) with increasing hydrogen content. In addition to the study on thermodynamic stability of these predicted structures, we have also investigated their dynamic stability, and the results indicate most of them are found to be recoverable when the pressure is partially or completely released. For example, the phonon calculations reveal no imaginary vibrational modes for the NaClH_2_ in *Pc* phase (Fig. S37a), KIH_2_ in the *P*6_3_/*mmc* structure (Fig. S14a), and RbI(H_2_)_2_ in the *Pmmn* structure (Fig. S14c) at ambient conditions.

We will investigate the chemical interaction between SM and the ionic sublattices by various electronic structure analysis methods, including a rigid band structure analysis, Bader's quantum theory of atoms in molecules (QTAIM) [24], electron localization functions (ELFs) [25], projected density of states (PDOS), and crystal orbital hamilton population (COHP) [26]. Our study will focus on NaCl-H_2_ compounds. The bonding features in all salt-SM compounds are quite similar.

**Figure 3.**
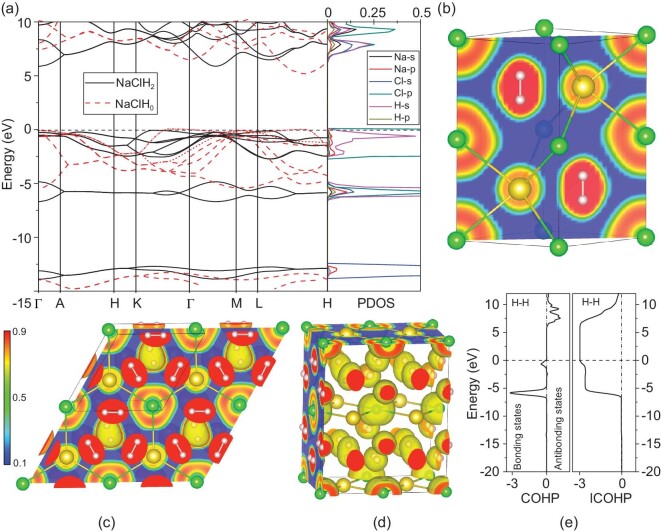
Calculated electronic properties for NaClH*_n_* at various pressures. (a) The electronic band structure and PDOS of *P*6_3_/*mmc* phase for NaClH_2_ at 20 GPa. In the left panel, the black solid lines are the electronic band structure of NaClH_2_; the red dashed lines are those of NaClH_0_ in which all the H_2_ molecules are removed from the NaClH_2_ structure. The black and red dashed lines show the Fermi energies of NaClH_2_ and NaClH_0_. The right panel presents the projected DOS of NaClH_2_. (b–d) The calculated ELFs of NaClH_2_ with *P*6_3_/*mmc* phase at 20 GPa, NaCl(H_2_)_4_ with *Pm* symmetry at 50 GPa, and NaCl(H_2_)_4_ with *Cmmm* symmetry at 100 GPa. (e) Calculated COHP and ICOHP of NaClH_2_ at 20 GPa.

The band structure of NaClH_2_ at ambient pressure shows a large energy band gap of nearly 5.6 eV (Fig. 3a). This value is close to the NaCl band gap under ambient pressure. In order to verify the effect of electroneutral H_2_ insertion on the electronic structure of the ionic sublattice, we also constructed a model system of NaClH_0_, in which all the H_2_ molecules are removed from the system. By comparing the band structures of NaClH_2_ with NaClH_0_, as shown in Fig. 3a, the results show that the H_2_ molecules do not significantly interfere with NaCl bands around the Fermi energy, which also suggests weak interactions between the H_2_ molecule and the ionic sublattice. Two groups of valence bands are heavily involved in H_2_ insertion in NaClH_2_, the upper one ranges from the Fermi level (0 eV) to −2.5 eV, and the lower one ranges from about −4.9 eV to −7.1 eV. After checking the projected components, we found that the upper groups are mainly the Cl *3p* orbitals, whereas the lower group are mainly the H *1s* orbitals. The bands in the upper group correspond to the Cl *3p* bands in NaClH_0_ (dashed red lines). The insertion of H_2_ into the NaCl lattice alters these bands to a considerable amount due to the occupation of the interstitial sites surrounded by Cl^−^ ions. On the other hand, the H_2_ bands only slightly overlap with Cl^−^ and Na^+^ states, indicating a weak interaction. This feature will be further proved by the following ELF and COHP calculations. Interestingly, the similar band structure and PDOS in all the salt-(H_2_)*_n_* and other NaCl(SM)*_n_* systems (Figs S18–S29) confirmed the weak interaction between NaCl and SM, implying the insertion nature of molecules in ionic compounds.

The Bader QTAIM charge [24] calculations (Table S1) support a model of NaCl ionic compounds inserted by neutral H_2_ molecules. The Bader charges on Na, Cl, and H atoms are found to be 0.88, −0.85, and 0.33 under 0 GPa, respectively. It is typical that the Bader charges are significantly smaller than the nominal charges. For example, the Bader charges of Na and Cl in NaCl crystal under 0 GPa are 0.82 and −0.82. For the same reason, the small residual charges found on H do not indicate the charge transfer between H_2_ and NaCl lattices. As a matter of fact, the insertions of He into NaCl and elemental Na also show a small charge of 0.83 under 50 GPa and of 0.81 under 100 GPa.

In conjunction with the Bader charge results, the ELF [25] calculations (Fig. 3b–d) reveal that inserted H_2_ molecules do not strongly bond with surrounding Na^+^ and Cl^−^ ions. The ELFs of *Pm* phase at 50 GPa (Fig. 3c) and *Cmmm* phases for NaCl(H_2_)_4_ at 100 GPa (Fig. 3d) unambiguously show the same bonding nature of Na–Cl and H–H. The low ELF values between Na and Cl confirm the ionic bonding nature (Fig. 3b), whereas the high ELF values between neighboring H atoms are in accordance with the fact that neighboring H atoms are covalently bonded and form H_2_ molecules. On the other hand, the low ELF values reveal very weak local bonding between H_2_ molecular units and the surrounding ions in both compounds. Similar results are found for other salt-SM hybrid compounds predicted in this work.

To further examine the bonding strength, we calculated the COHPs [26] and the integrated COHPs (ICOHP) between neighboring atoms in these compounds. The results show the full occupation of the H–H bonding states and the empty H–H antibonding states, revealing that the bonding feature of H_2_ molecules is not largely influenced while inserted into the NaCl crystal (Fig. 3e). For comparison, we calculated the ICOHP values up to the Fermi level for H–H pairs in NaCl-H_2_ compounds and in solid hydrogen at various pressures. The ICOHP value of −2.9 pairs/eV of the H–H bond reveals the strength of H–H in salt-H_2_ systems. This is also in conformity with the fact that the bond length (0.72 Å) of H_2_ in salt-H_2_ is close to that (0.74 Å) in pure solid H_2_.

**Figure 4.**
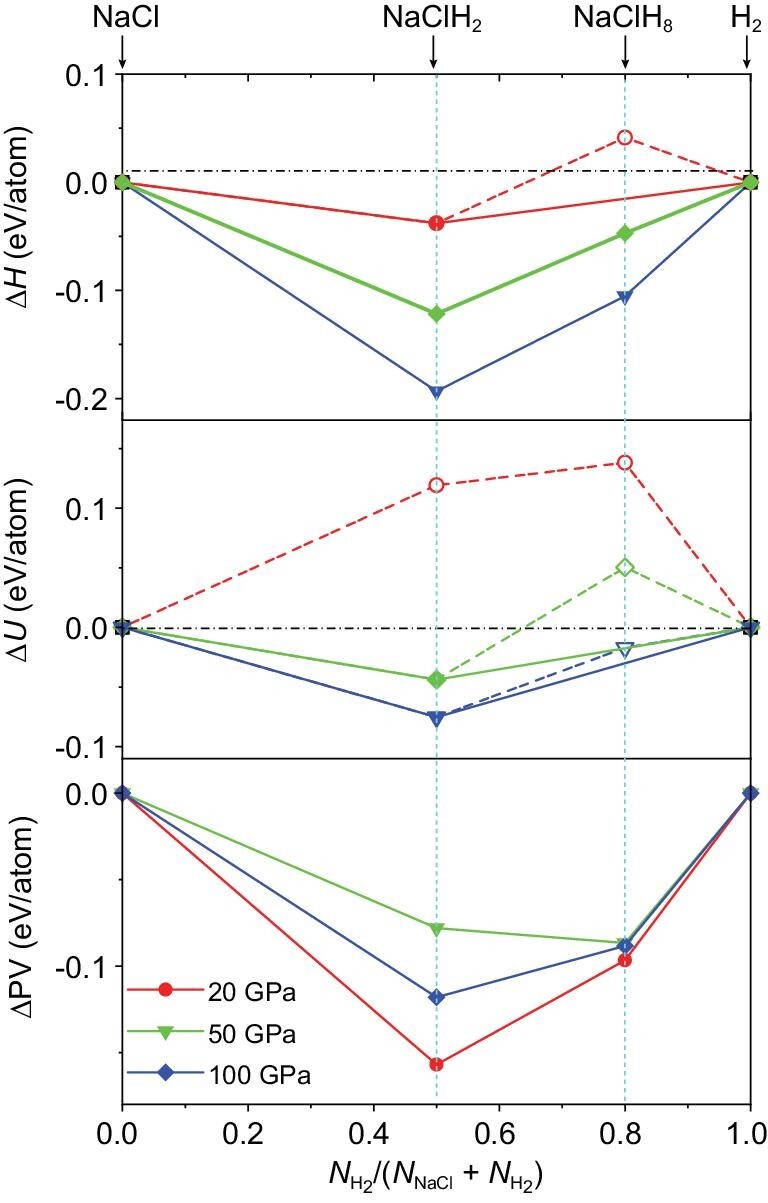
Energy contributions to the formation of NaCl-H_2_ hybrid compounds. Reaction enthalpies (top panel), internal energy changes (middle panel), and PV term changes (bottom panel) for the formation of NaCl-H_2_ hybrid compounds at different pressures.

If the inserted molecules do not interact strongly with the surrounding ions, what mechanism drives the formation of these unusual hybrid compounds under pressure? We thoroughly investigate the origin of the thermodynamic stability of the salt-SM compounds, starting from the split of the reaction enthalpies of NaCl-H_2_ compounds as shown in Fig. 4, ∆*H*, into the internal energy changes ∆*U* and the pressure-volume terms ∆PV.

For NaClH_2_, although ∆*U* is positive at low pressure whereas ∆PV is negative, both terms decrease with increasing pressure, contributing to the continuous decrease of ∆*H*, leading therefore to the stabilization of the hybrid compound. In particular, the ∆PV term decreases much more significantly than ∆*U*, indicating that the formation of XY-H_2_ hybrid compounds is primarily driven by volume reduction. For compounds with higher hydrogen composition, such as NaCl(H_2_)_4_, the decrease in ∆PV is less significant. However, it still makes major contributions to the reaction enthalpy and the stabilization of inserted compounds. It is worth noting that many recently predicted and synthesized metal hydrides, such as NaH*_n_* [27], LiH*_n_* [28] *etc*, contain H_2_ molecules in their crystal lattices. Many of these hydrides can be viewed as XH-H_2_ hybrid compounds, further demonstrating that the small molecule inclusion is an extensive phenomenon under high pressure.

Most other molecules, such as H_2_O, NH_3_, CH_4_, and CO_2_, behave similarly to H_2_ while inserted into NaCl, i.e. they do not bond strongly with the neighboring Na^+^ and Cl^−^ ions. In contrast, while searching low enthalpy structures of N_2_-inserted NaCl, we found a striking phenomenon, namely, N_2_ molecules will decompose in NaCl and form pentazolate anions (cyclo-N_5_^−^) (Fig. 2d) inserted into the NaCl crystal at pressures ranging from 36 to 83 GPa (Fig. S16a). Calculated ICOHP values for the N–N bond at 50 GPa is −1.56 eV/pair, indicating that there is a strong covalent bond of N–N in cyclo-N_5_^−^ units (Fig. S16c). The Bader QTAIM calculations show charges of 0.86, 0.28, and −0.114 for Na, Cl, and N atoms, which suggest a large charge transfer from Cl^−^ to cyclo-N_5_^−^. Therefore, the formation of NaCl(N_5_)_2_ is due to the oxidation of Cl^−^ by cyclo-N_5_^−^. The band structure and PDOS of *C*2/*c*-NaCl(N_5_)_2_ at 50 GPa (Fig. S16b) show that the electron states of the cyclo-N_5_^−^ contribute to both valence and conduction bands in a large energy range around the Fermi level and overlap with states of Na and Cl. Pentazolates are promising candidates for high-energy-density materials that do not release harmful products upon decomposition. However, its syntheses are hindered by low stability and usually involve additions of metal and organic stabilizers, forming complex structures. Our research shows that the commonly known ionic compounds such as NaCl that are easy to obtain and handle can be used to react directly with N_2_ under pressure and produce compounds containing large compositions of pentazolate anions. As a matter of fact, the predicted NaCl(N_5_)_2_ structure has nearly 71% weight ratio of nitrogen in the form of cyclic N_5_ units. The ambient-pressure decomposition of NaCl(N_5_)_2_ is estimated to possibly release 3.78 kJ·g^−1^ energy per NaCl(N_5_)_2_ unit. Moreover, our molecular dynamics simulations confirm the metastable feature of this predicted structure at ambient conditions (Fig. S17). The present results may open a new avenue to discover high-energy-density materials of polynitrogen compounds.

Although the crystal structure search predicts that H_2_O molecules insert in NaCl similarly to H_2_, the potentially stronger interactions between the H_2_O molecule and surrounding ions merit an in-depth analysis of its electronic structure. As shown in Fig. 1 and Fig. S16d, the predicted monoclinic structure of (NaCl)_2_H_2_O with *Pnma* symmetry (Fig. 2e) can become stable at a high pressure of 102 GPa. Furthermore, the band structure and PDOS for the *C*2/*c* structure at 200 GPa (Fig. S16e) show that the entire energy range is dominated by the bands of the Cl anion. A large band gap of nearly 4.2 eV appears between the valence and conduction bands. The calculated COHP (Fig. S16f) clearly shows that the O–H bonding states are occupied, whereas the antibonding states are not. Correspondingly, the ICOHP value with −1.0 eV/pair of O–H bonds supports the strong covalent nature of O–H covalent bonding in H_2_O molecules. Despite the stronger interactions with the surrounding ions, H_2_O maintains its major molecular bonding features while inserted into NaCl.

Our results provide an important piece of knowledge for the understanding of the interior structure and dynamics of planets. Uranus and Neptune are called ‘ice giants’ in our solar system because they contain significant amounts of icy materials (mainly H_2_O, CH_4_, and NH_3_). In addition, abundant ionic compounds exist in the rocky layer of Mars, Venus, Uranus, and Neptune. It is widely accepted that most exoplanets may assume similar multi-shell structures consisting of layers of molecular and ionic rocky materials. Thus, the molecule–rock interaction and its properties under pressure may govern the inter-layer structures of most intermediate-mass exoplanets. While our study demonstrates that typical ionic compounds such as NaCl can react with icy materials, the general chemistry it reveals can potentially be extended to other compounds, including the rock materials, such as binary and ternary oxides, in the planet's interior. Indeed, recent works uncovered that magnesium oxide–water compounds could become stable at high pressure and offered a renewed understanding of planetary interiors [29].

## CONCLUSIONS

In conclusion, we demonstrate a unique chemical phenomenon under pressure using the first-principles calculations and the crystal structure search method: many covalently bonded molecules can form stable compounds by mixing into ionic crystals while maintaining their molecular integrity. The phenomenon is manifested in many striking predictions. For example, H_2_ is predicted to react with NaCl to form NaClH_2_ and NaCl(H_2_)_4_ at 20 and 38 GPa pressures, respectively. A similar reaction could happen at a much lower pressure of 1 GPa if H_2_ is inserted into potassium iodide (KI), which facilitates future experimental synthesis and applications by bringing the reaction pressure down to the sub-GPa level. Similar insertions are also found for heteronuclear molecules such as H_2_O, NH_3_, and CH_4_, despite whether the molecule is polar or non-polar. The crystal structure search also predicted the insertion of C_2_H_6_ into NaCl under high pressure, indicating the wide range of the phenomenon and the possibility that large organic molecules might be chemically reserved inside the rocky interior of planets. Last, to our surprise, the most stable one among all molecules in this work, N_2_, is found to transform chemically while inserted into NaCl and become cyclo-N_5_^−^ which oxidizes NaCl. This reaction could become a new route to synthesize and stabilize pentazolates, green energy materials with high energy density. Our results suggest that the molecular and rock layers in the interiors of large planets may exhibit large regions in which the molecules and solid compounds diffuse into each other and are chemically mixed.

## METHODS

### Crystal structure prediction

Our structure searching simulations are performed through the swarm-intelligence based CALYPSO method [18] via a global minimization of free energy surfaces merging *ab initio* total-energy calculations as implemented in the CALYPSO code [19] and random structure searching as implemented in the AIRSS code [30,31]. These methods are specially designed for unbiased global structural optimization, and have been benchmarked on various known systems [31–34].

### Total energy calculations

Total energy calculations were performed in the framework of density functional theory within the Perdew-Burke-Ernzerhof [35] parameterization of generalized gradient approximation [36] as implemented in the VASP (Vienna Ab Initio simulation package) code [37]. The projector-augmented wave (PAW) method [38] was adopted with the PAW potentials taken from the VASP library where *d* electrons are treated as valence electrons for alkali elements. The use of the plane-wave kinetic energy cutoff of 1200 eV (for H_2_ molecules) and 800 eV (for NH_3_, H_2_O, CH_4_, CO_2_, N_2_ molecules) and dense k-point sampling, adopted here, were shown to give excellent convergence of total energies (within ∼1 meV/atom). We explored the zero-point energy effects on the formation energy using the phonopy code [39].

## Supplementary Material

nwae016_Supplemental_File
