# Supplementary material for: Universal insertion of molecules in ionic compounds under pressure
Source: Natl Sci Rev. 2024 Jan 11;11(1):nwae016. doi: 10.1093/nsr/nwae016 (PMC10858741; doi:10.1093/nsr/nwae016)
Supplement: nwae016_Supplemental_File [file nwae016_supplemental_file.pdf]

Supplementary information

of

Universal insertion of molecules in ionic compounds under pressure

Feng Peng,[1,2] Yanming Ma,[3,4] Chris J. Pickard[5,6], Hanyu Liu,[3,4*] and Maosheng Miao[2,7]*

[1]*College of Physics and Electronic Information, Luoyang Normal University, Luoyang 471022, China*
[2]*Department of Chemistry and Biochemistry, California State University Northridge, Northridge, CA, 91330-8262, USA*
[3]*State Key Laboratory of Superhard Materials & Key Laboratory of Material Simulation Methods and Software of Ministry of Education, College of Physics, Jilin University, Changchun 130012, China*
[4]*International Center of Future Science, Jilin University, Changchun 130012, China*
[5]*Department of Materials Science & Metallurgy, University of Cambridge, 27 Charles Babbage Road, Cambridge CB3 0FS, United Kingdom*
[6]*Advanced Institute for Materials Research, Tohoku University 2-1-1 Katahira, Aoba, Sendai, 980-8577, Japan*
[7]*Department of Earth Science, University of California Santa Barbara, USA*

*Correspondence Email: M. M. (mmiao@csun.edu) and H. L. (hanyuliu@jlu.edu.cn)

Computational details

Our structural prediction approach is based on a global minimization of free energy surfaces merging ab initio total-energy calculations through CALYPSO (Crystal structure AnaLYsis by Particle Swarm Optimization) methodology as implemented in its same-name CALYPSO code [1,2]. The effectiveness of our CALYPSO method has been demonstrated by the successful applications in predicting high-pressure structures of various systems, ranging from elements to binary and ternary compounds[1]. Particularly, high-pressure structures of lithium [3], bismuth telluride [4], water ice [5], calcium hydride [6], cage-like diamondoid nitrogen [7], and xenon-iron compounds[8] were predicted, among which the high-pressure insulating *Aba*2-40 (Pearson symbol *oC*40) structure of lithium and the two low-pressure monoclinic structures of bismuth telluride were confirmed by independent experiments [9], guided by our theoretical predictions of the synthetic precursors, temperatures, pressure ranges and pathways corresponding to the stable structures. Ab Initio Random Structure Searching (AIRSS) [10] calculations were also employed to confirm some of our key structures, while some H-richer stoichiometries XYH_n (n =12-20) were also considered in the AIRSS approach [10].

Structure searches for hybrid compounds formed by NaCl and small molecules of $NaCl_x(SM)_y$ (x, y = 1-4) and alkali halides XY hydrides of XYH_n (n = 1 – 20) with simulation cells ranging from one to four formula units were performed at up to 200 GPa. Each generation contained 50 structures, and structures in the first generation were produced randomly with symmetry constraints. All the generated structures are optimized to their local minima by using DFT calculations. Here, almost all geometrical optimization calculations are performed by using PAW potentials and VASP code [11] and CASTEP code[12]. During structure evolution, 60% of structures in the first generation with lower enthalpies are selected to produce the structures in the next generation by PSO. 40% of the structures in the new generation are randomly generated. A structure fingerprinting technique of bond characterization matrix is applied to produce structures, so that identical structures are strictly forbidden. These procedures significantly enhance the diversity of structures, which is crucial for the searching efficiency of algorithm. The local optimizations are performed by use of the conjugate gradients method and the criteria of the enthalpy change is 1×10^{-3} eV per atom. For most of the cases, the structure searches reach the convergence after 50 generations covering about 2500 structures.

Once we have the predicted structures at hand, we then optimized a number of low-enthalpy structures at a higher accuracy as a function of pressure using either VASP method. The plane wave

basis set was constructed using the energy cutoff of 1200 eV (for H_2 molecule) and 800 eV (for NH_3, H_2O, CH_4, CO_2, N_2 molecules) in all calculations, and the Brillouin zone was sampled with a k-point resolution of $2\pi \times 0.03$ Å^{-1}. This usually gives total enthalpies well converged within ~ 1 meV/atom. The PAW potentials [13] are adopted in the VASP calculations. The exchange-correlation functional was described using Perdew–Burke–Ernzerhof [14] of generalized gradient approximation [15].

Based on the analysis of a quantum mechanical observable, electronic density, while taking proper account of the influence of Pauli's exclusive principle, Becke and Edgecombe introduced the Electron Localization Function (ELF) [16] defined so as to have the convenient range of values $0 <$ ELF < 1. Regions in which the value of ELF is close to 0.5 or 1.0 correspond to perfect free-electron gas distribution or well-localized electrons, respectively. This approach leads to clear quantitative demarcation between covalent and non-covalent bonding. As a result, the ELF has been widely used to describe and visualize chemical bonds in molecules and solids.

The phonon calculations were carried out by using supercell approach [17] as implemented in the phonopy code [18]. All structures of NaCl-SM compounds are demonstrated to be dynamically stable in their stable pressure ranges by phonon calculations.

The calculation of the electron population was performed using the Bader Charge Analysis code developed by the Henkelman group in the University of Texas at Austin.

Supplemental Figures

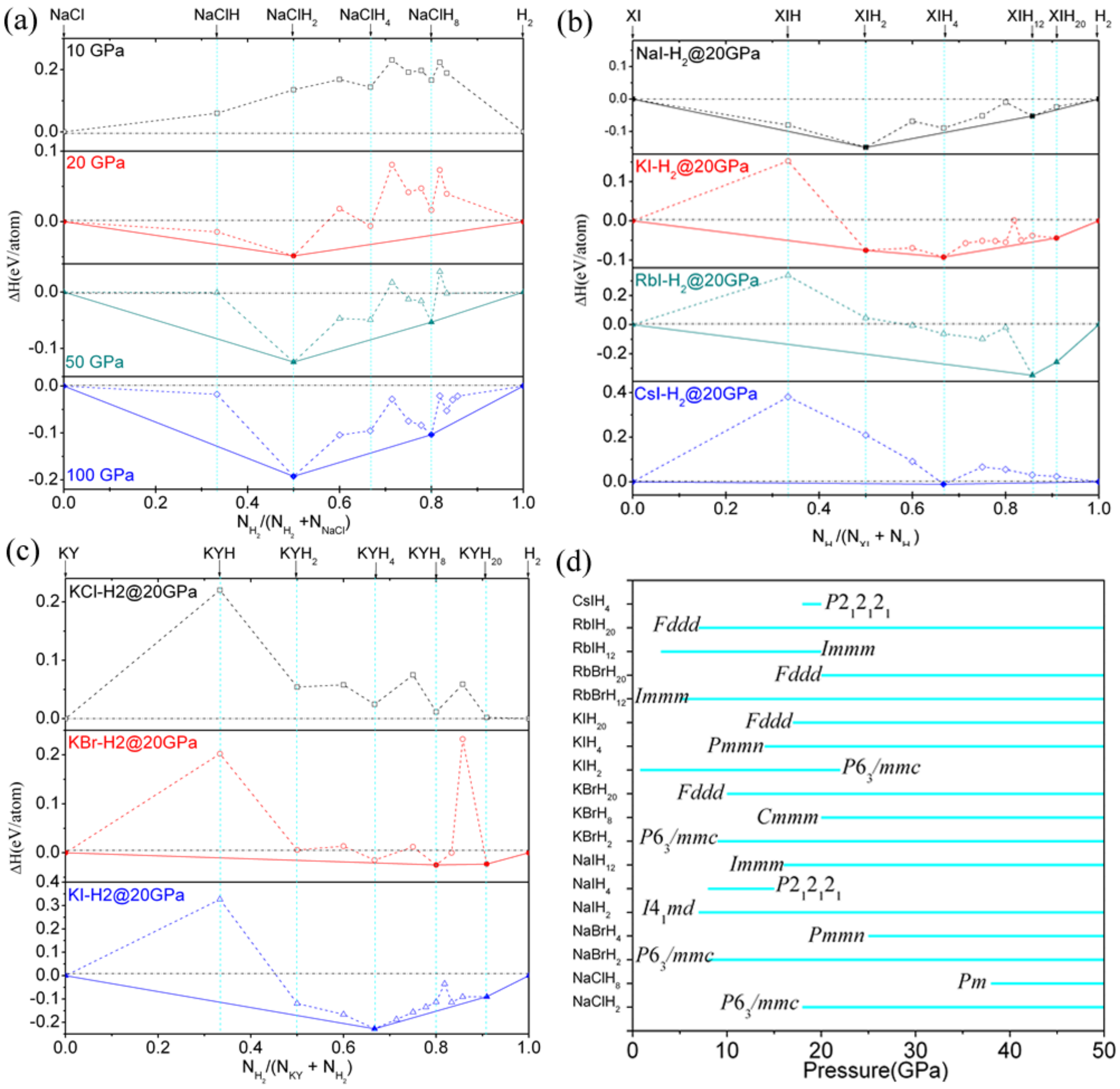

Figure S1. Phase stabilities of various H-rich XY-H_2 hybrid compounds. Enthalpies of formation of (a) NaCl-H_2 under several pressures up to 100 GPa; (b) XY-H_2 (X=Na, K, Rb, Cs; Y=I) under 20 GPa; (c) KY-H_2 (Y=Cl, Br, I) under 20 GPa. Dotted lines connect the data points, and solid lines denote the convex hull. (d) Predicted pressure-composition phase diagram of XY salt hydrides.

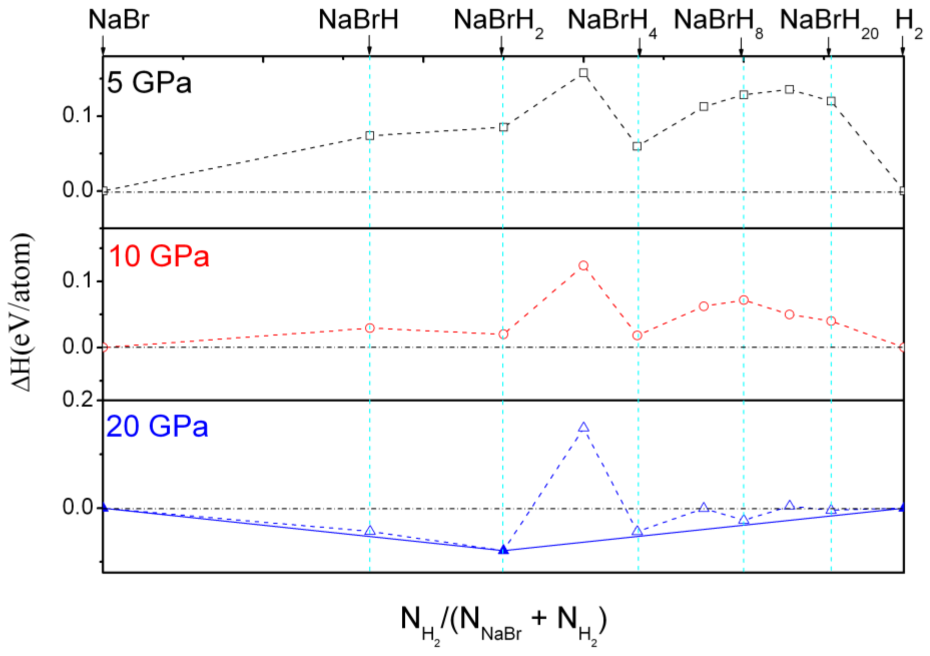

Figure S2. Enthalpies of formation of various H-rich NaBr hydrides under pressures. The dotted lines connect the data points, while the solid lines denote the convex hull. $NaBrH_2$ is the most stable species for all pressures studied.

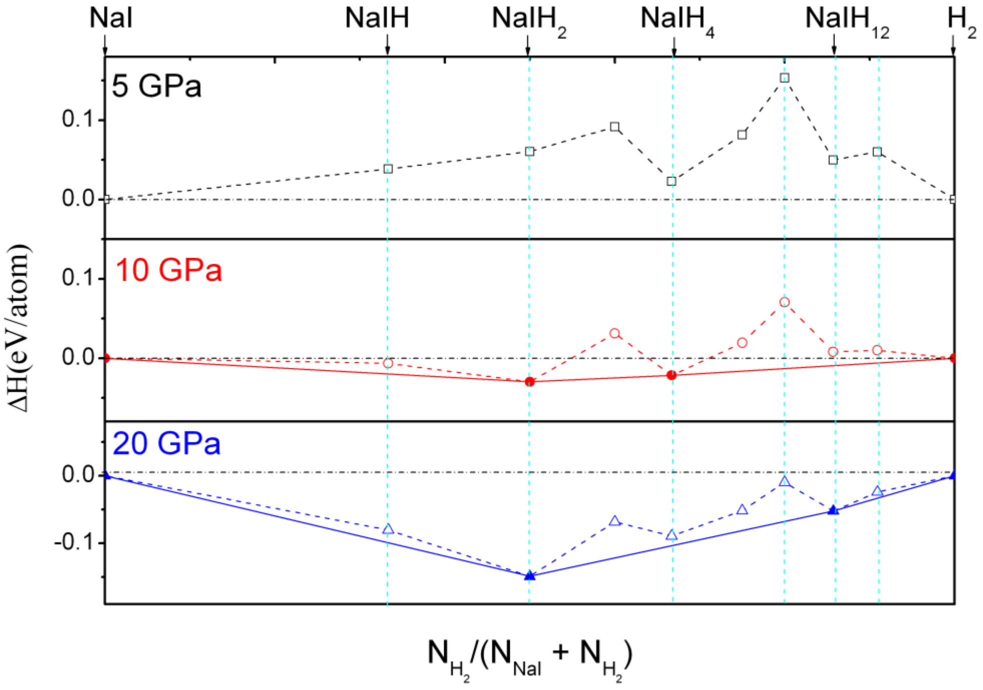

Figure S3. Enthalpies of formation of various H-rich NaI hydrides under pressures. At P = 5 GPa, the Enthalpies of formation (h_f) values of all stoichiometries are positive, and no stoichiometries can be stable. $NaIH_2$ is the most stable at the pressure rang of 10-20 GPa.

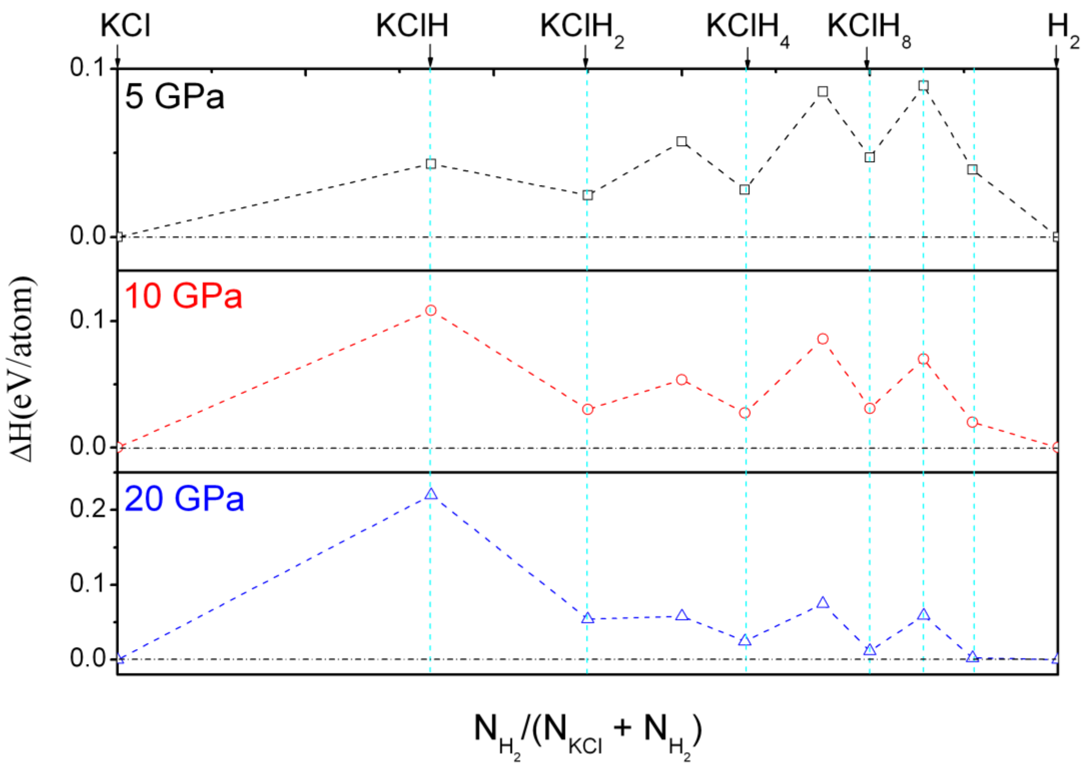

Figure S4. Enthalpies of formation of various H-rich KCl hydrides under pressures. In the pressure range of 5-20 GPa, the Enthalpies of formation (h_f) values of all stoichiometries are positive, and no stoichiometries can be stable.

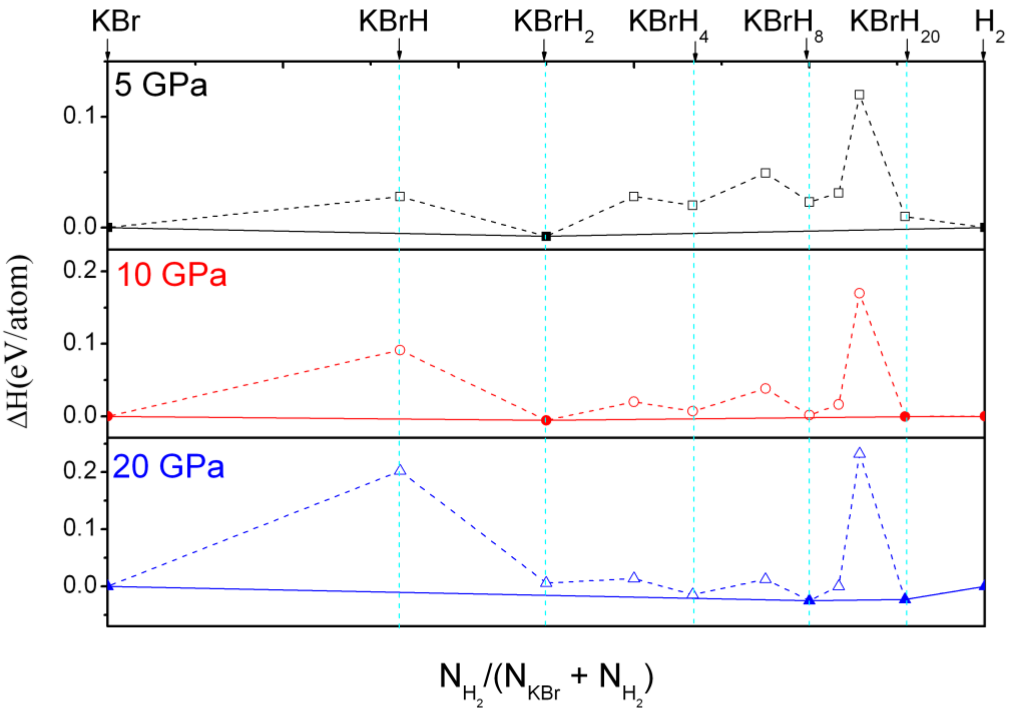

Figure S5. Enthalpies of formation of various H-rich KBr hydrides under pressures. $KBrH_2$ is the most stable phase in the pressure range of 5-10 GPa, while $KBrH_8$ turns out to be most stable at 20 GPa.

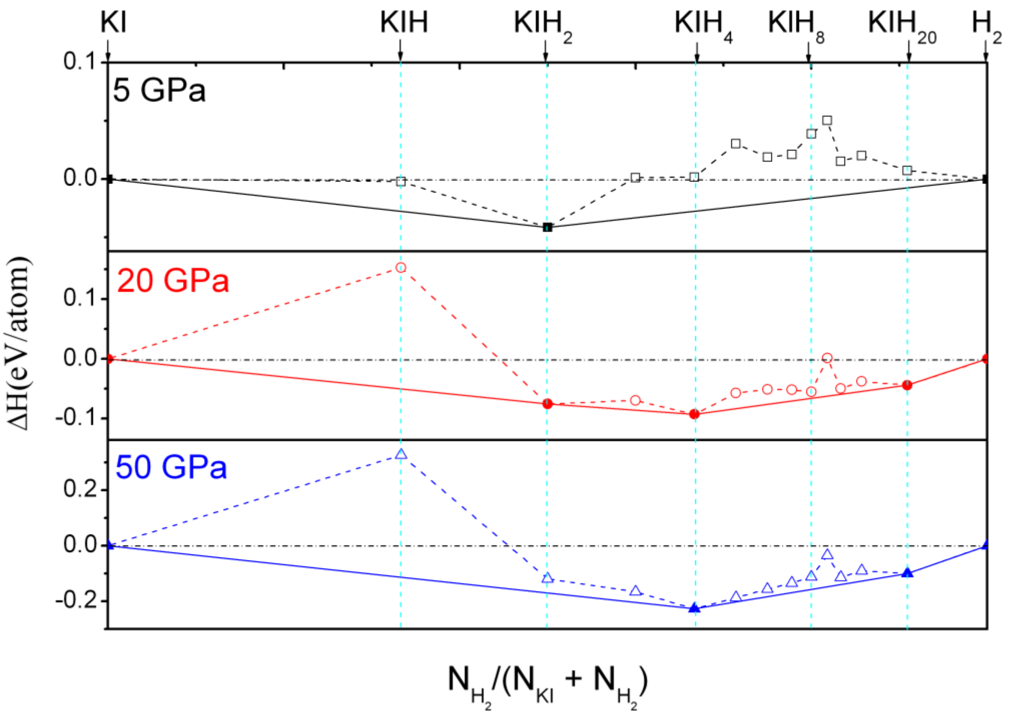

Figure S6. Enthalpies of formation of various H-rich KI hydrides under pressures. KIH_2 is the most stable phase at the pressures of 5 GPa, while KIH_4 is the most stable phase in the pressure rang of 20-50 GPa. At 20 GPa, $KBrH_2$, $KBrH_4$ and $KBrH_{10}$ are three stable phases, while at 50 GPa, $KBrH_4$ and $KBrH_{20}$ are stable.

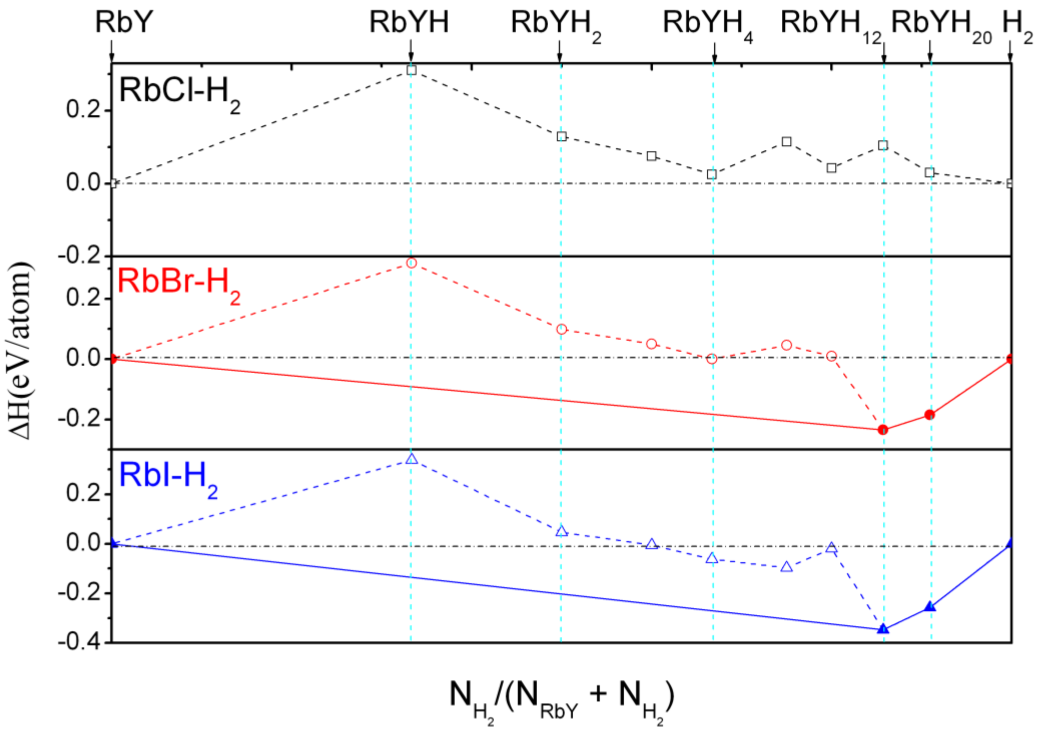

Figure S7. Enthalpies of formation of various H-rich RbY (Y are halogens) hydrides at 20 GPa. $RbYH_4$ is the most stable phase at 20 GPa. The stable pressure of $RbYH_4$ is reducing with increasing atomic number of the halogen elements.

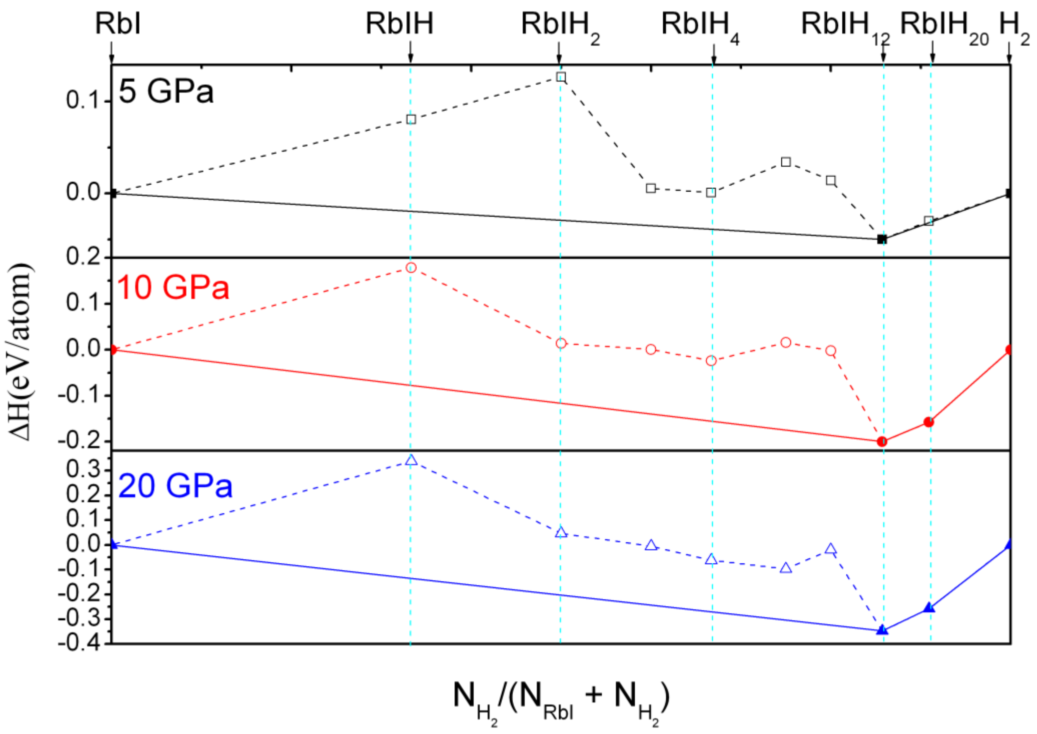

Figure S8. Enthalpies of formation of various H-rich RbI hydrides under high pressure. $RbIH_{12}$ is the most stable phase in the pressure range of 5-20 GPa.

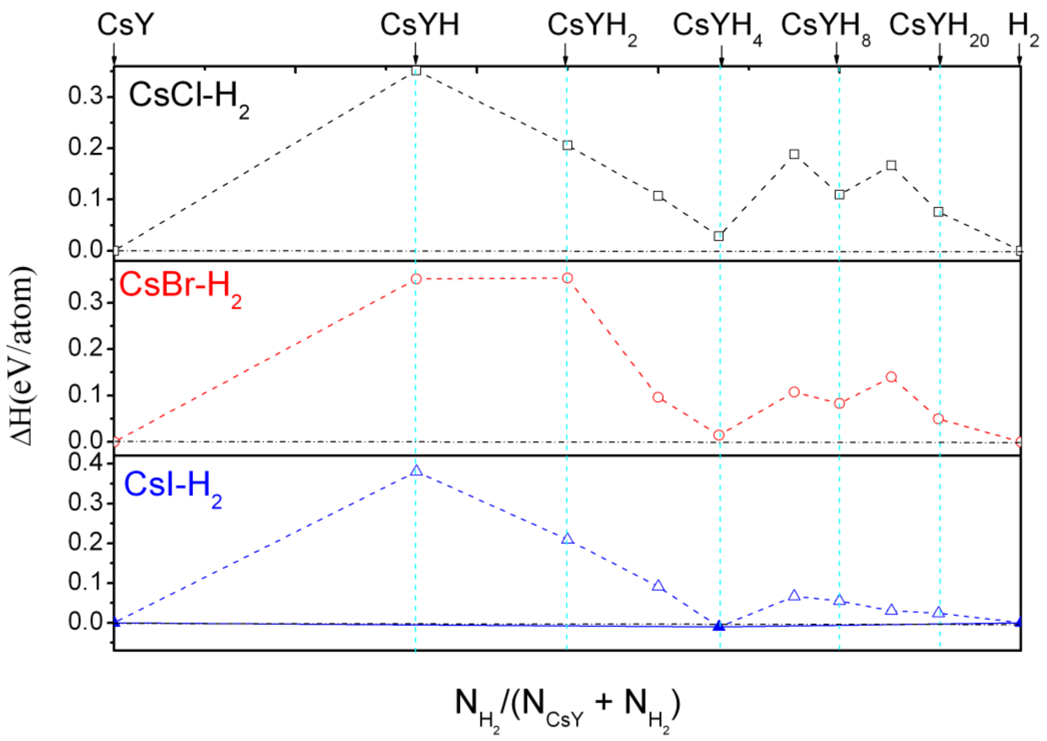

Figure S9. Enthalpies of formation of various H-rich CsY (Y are halogens) hydrides at 20 GPa. $CsYH_4$ is the most stable phase at 20 GPa. The stable pressure of $CsYH_4$ is reducing with increasing atomic number of the halogen elements.

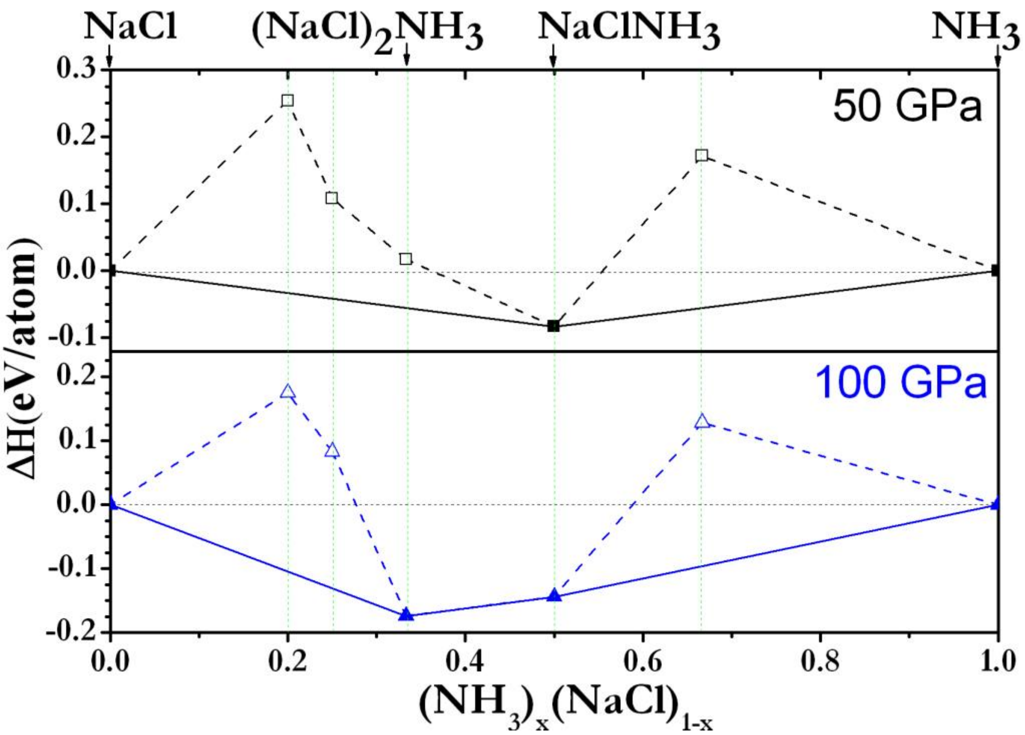

Figure S10. Enthalpies of formation of various NaCl-NH_3 compounds at 50 and 100 GPa. NaCl(NH_3) is the most stable phase at 50 GPa. Besides NaCl(NH_3), (NaCl)2(NH_3) becomes most stable at 100 GPa.

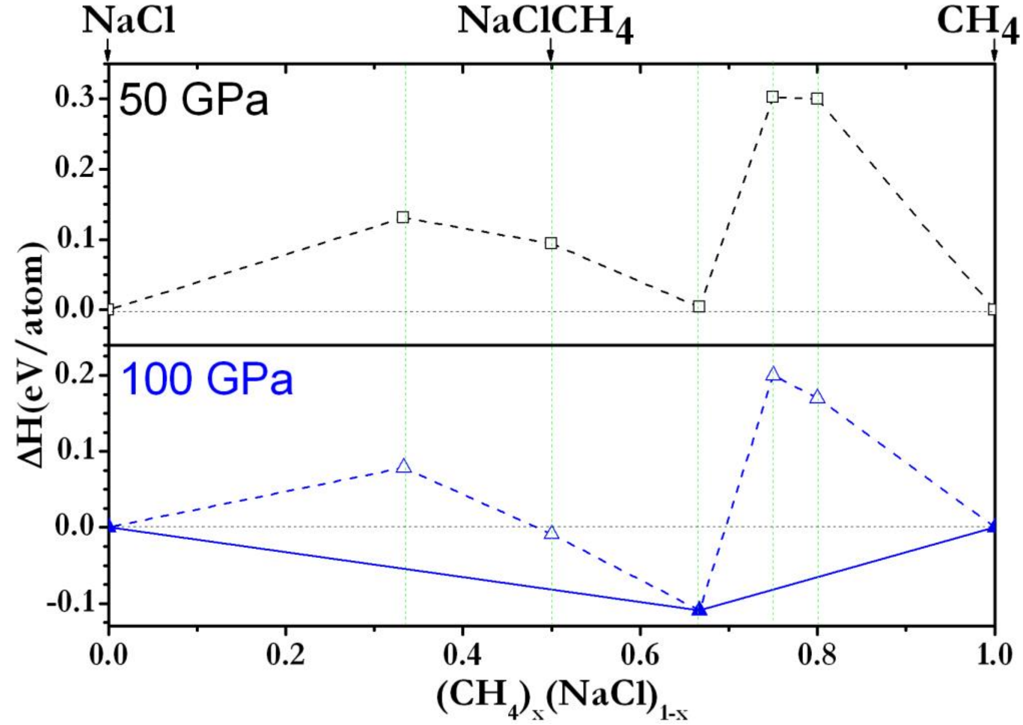

Figure S11. Enthalpies of formation of various NaCl-CH_4 compounds at 50 and 100 GPa. The formation enthalpies of all the stoichiometries are positive at 50 GPa, indicating they are all thermodynamically unstable at the pressure range of 0-50 GPa. NaCl$(CH_4)_2$ becomes stable at 100 GPa.

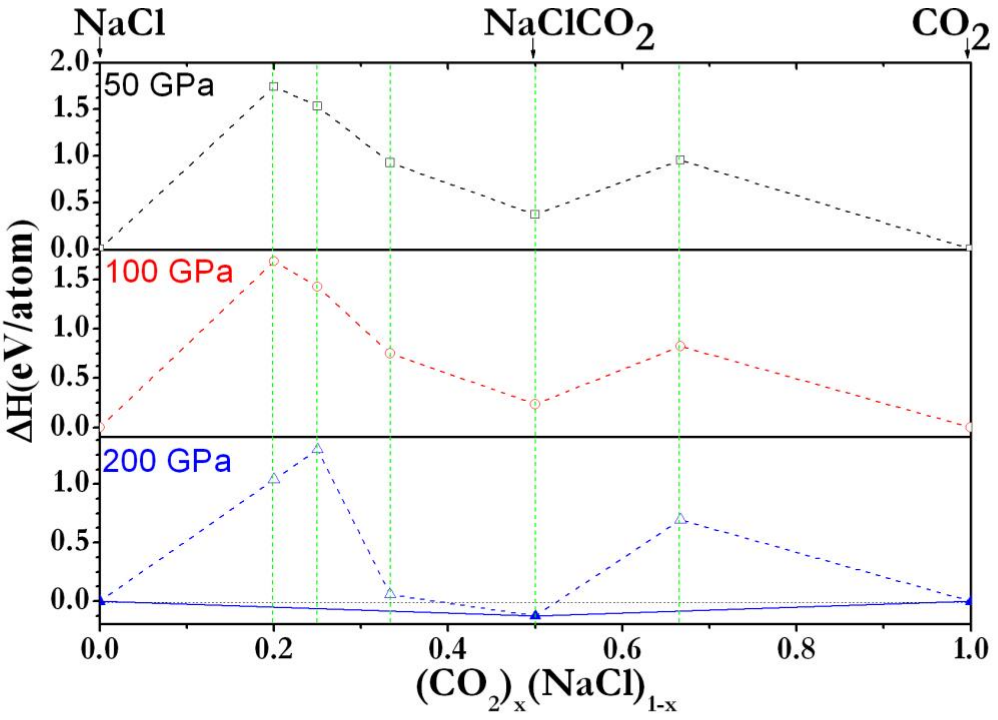

Figure S12. Enthalpies of formation of various NaCl-CH_4 compounds at 50, 100 and 200 GPa. The formation enthalpies of all the stoichiometries are positive at 50 and 100 GPa, indicating they are all thermodynamically unstable at the pressure range of 0-100 GPa. NaCl(CO_2) becomes stable at 200 GPa.

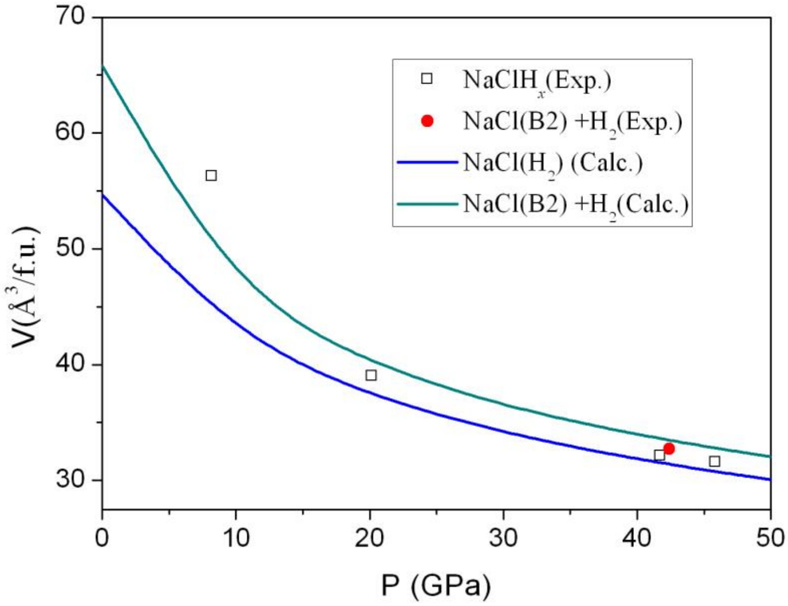

Figure S13. The equation of state of NaCl(H_2) and NaCl+H_2. Volumes (Å^3/f.u.) for the observed NaClH_x, the predicted NaCl(H_2) with $P6_3/mmc$ symmetry, and NaCl+H_2 vary with pressures.

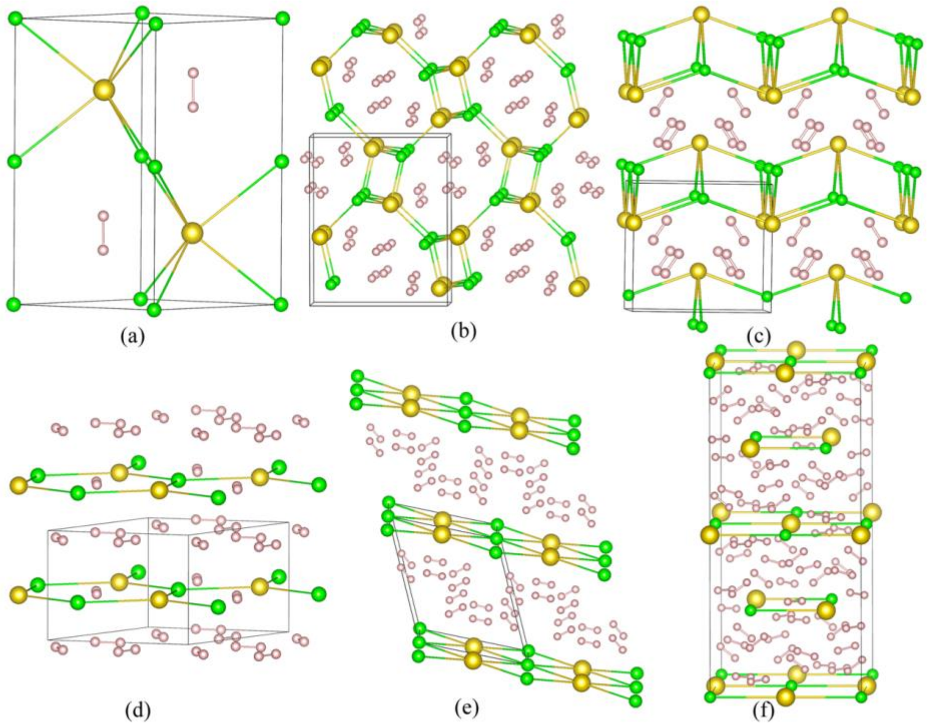

Figure S14. Structures of XYH*n* compounds. Three different ways of inserting H_2 molecules inside the lattices of the XY ions: at atomic sites in $P6_3/mmc$ symmetry (a); inside tubes in $XY(H_2)_2$ with $P2_12_12_1$ symmetry (b); and between layers in $XY(H_2)_2$ with *Pmmn* symmetry (c), $XY(H_2)_4$ with *Pm* symmetry (d), $XY(H_2)_6$ with *Immm* symmetry (e); and between linear chains in $XY(H_2)_{10}$ with *Fddd* symmetry (f). The white, gold and green spheres represent H, alkali (Na, K, Rb, Cs) and halogen (Cl, Br, I) atoms, respectively.

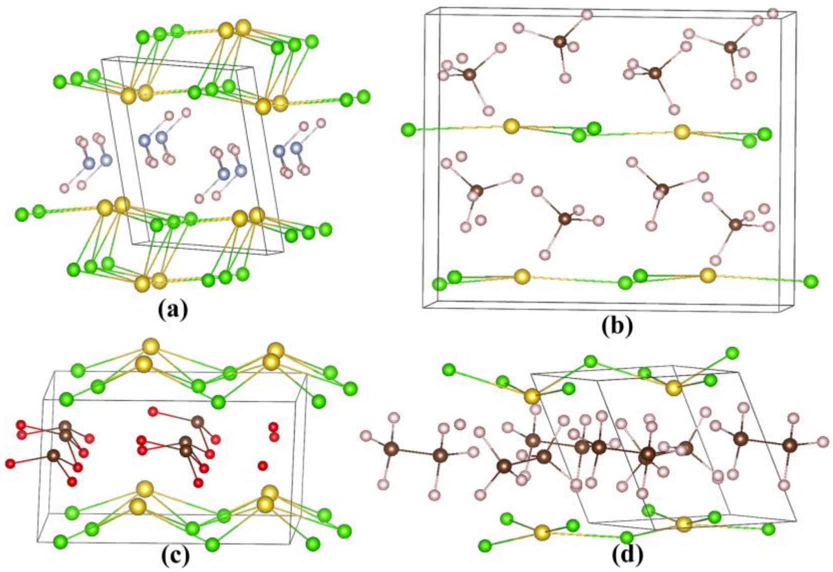

Figure S15. Structures of NaCl-SM compounds. Three different ways of inserting small molecules inside the lattices of the NaCl ions: NH_3 molecules between layers in $NaClNH_3$ with *P*-1 symmetry (a), $NaCl(CH_4)_2$ with *Aba*2 symmetry (b), $NaClCO_2$ with *Cm* symmetry (c); and $NaCl(C_2H_6)$ with *P*1 symmetry (d). The brown blue, pink, red, gold and green spheres represent C, N, H, O, Na and Cl atoms, respectively.

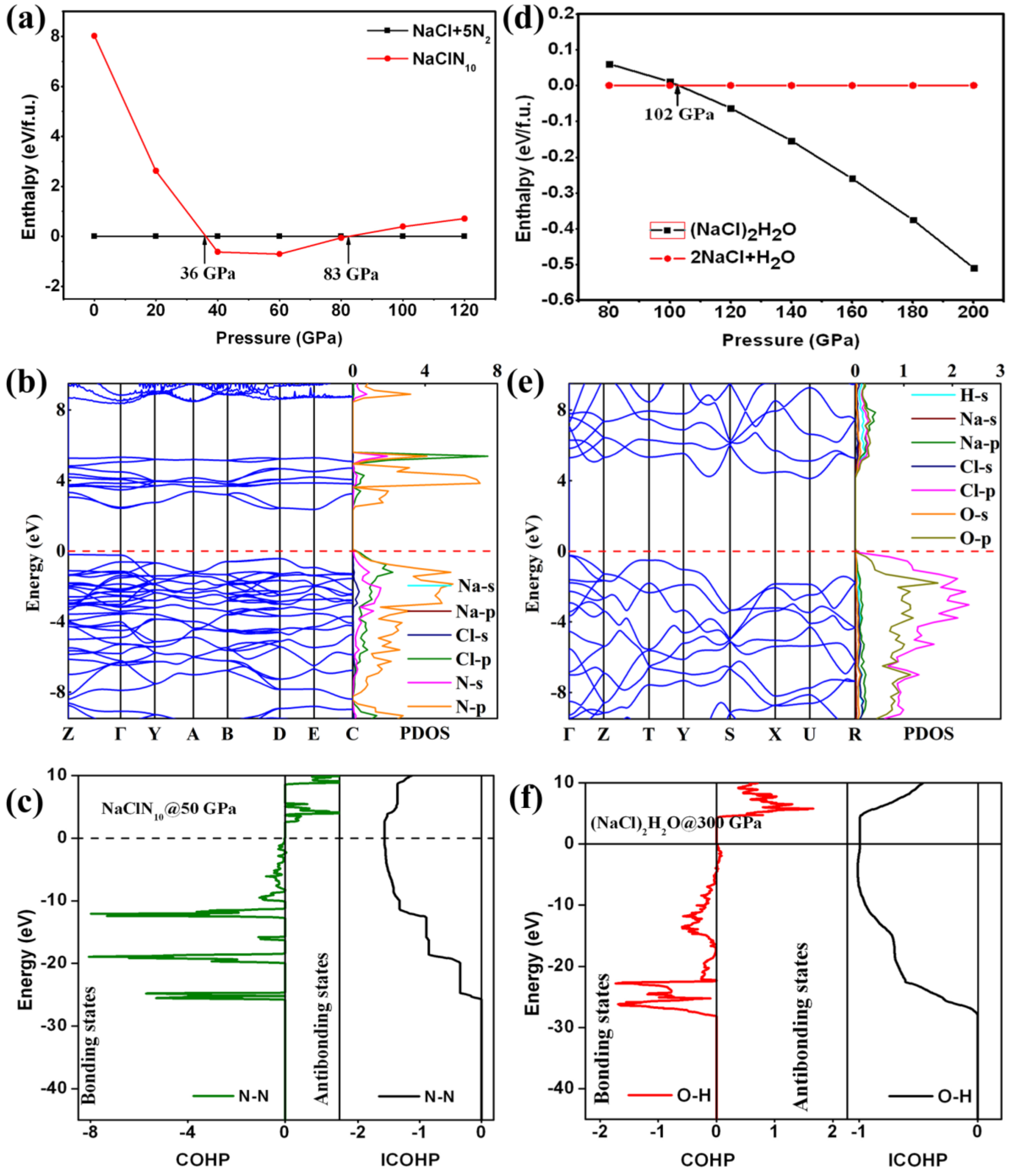

Figure S16. Calculated formation enthalpy and electronic properties for $NaCl(N_5)_2$ and $(NaCl)_2H_2O$ at high pressures. Calculated enthalpies as a function of pressure for $NaCl(N_5)_2$ (a) and $(NaCl)_2H_2O$ (d) with respect to NaCl and SM. The calculated electronic band structure and PDOS for $NaCl(N_5)_2$ (b) and $(NaCl)_2H_2O$ (e) at high pressures. Calculated COHP and ICHOP of N-N in $NaCl(N_5)_2$ (c) and H-O in $(NaCl)_2H_2O$ (f) at high pressures. In Figure S16b, we have calculated the energy band structure and projected DOS of *C*2/*c*-$NaCl(N_5)_2$ at 50 GPa, the calculation results show that the entire energy range is mainly contributed by the N_5 units. Calculated ICOHP values for the N-N bond at 50 GPa is -1.56 eV/pair indicated that there is a strong covalent bond between N-N in N_5 unit (Figure S16c).

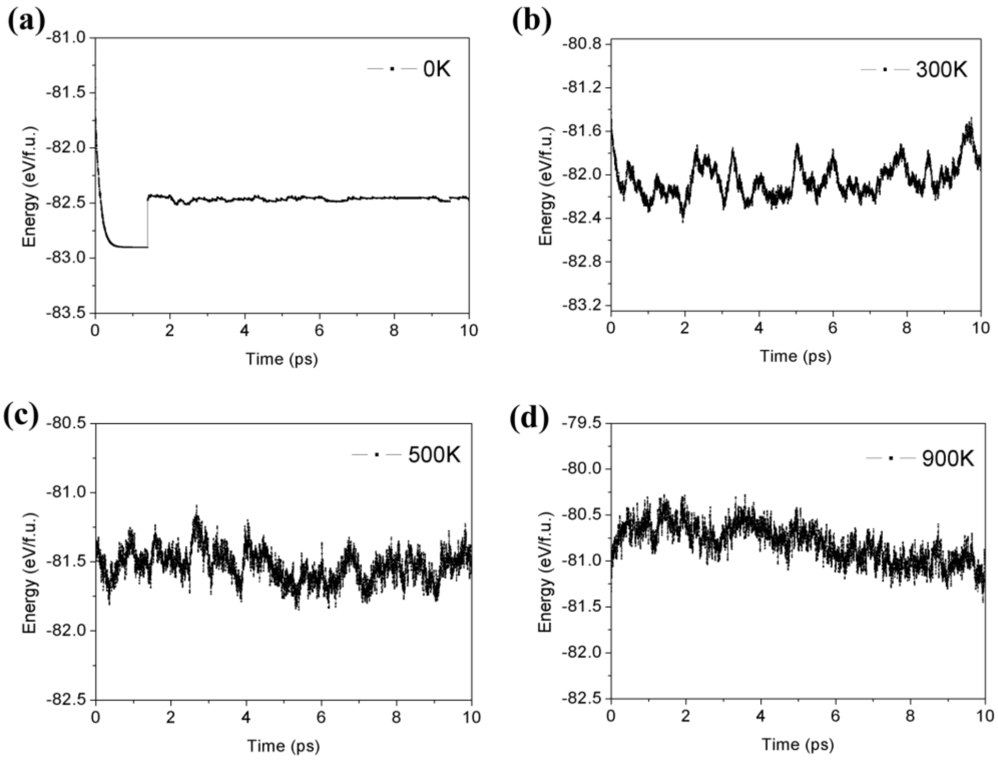

Figure S17. Enthalpy evolution during the molecular dynamics simulations at T = 0, 300, 500 and 900 K and ambient pressure. After typically long simulation runs (10 ps for $NaCl(N_5)_2$), the structure remains stable without having any obvious structural changes.

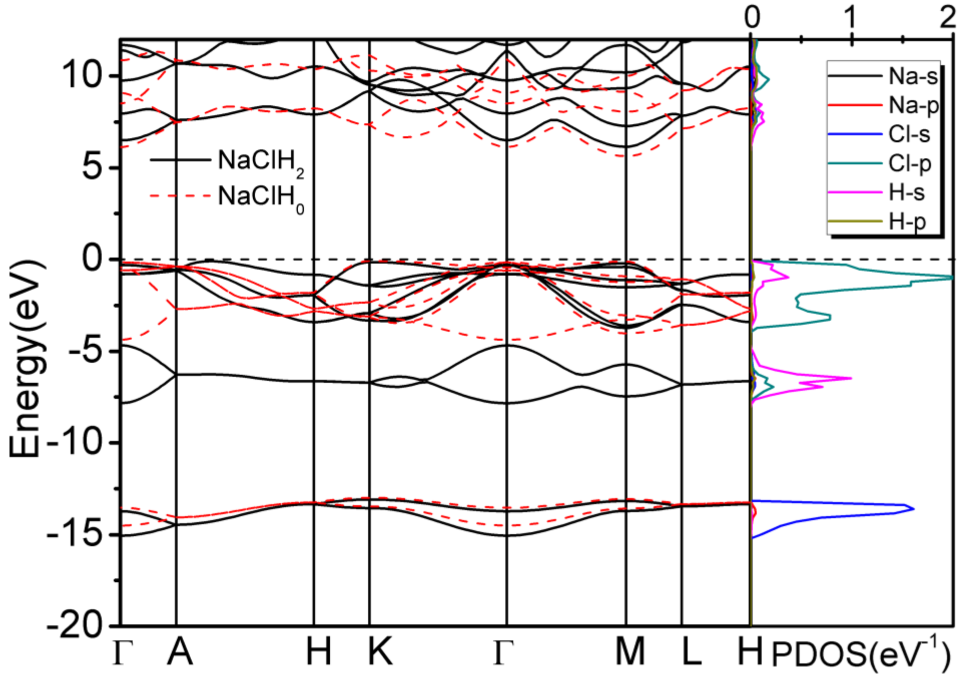

Figure S18. The electronic band structure and PDOS of *P*6₃/*mmc* phase for $NaClH_2$ at 0 GPa. In the left panel, the black solid lines are electronic band structure of $NaClH_2$; the red dashed lines are those of $NaClH_0$ in which all the H_2 molecules are removed from the $NaClH_2$ structure. The black line shows the Fermi energy of $NaClH_2$ and $NaClH_0$. The right panel presents the projected DOS of $NaClH_2$.

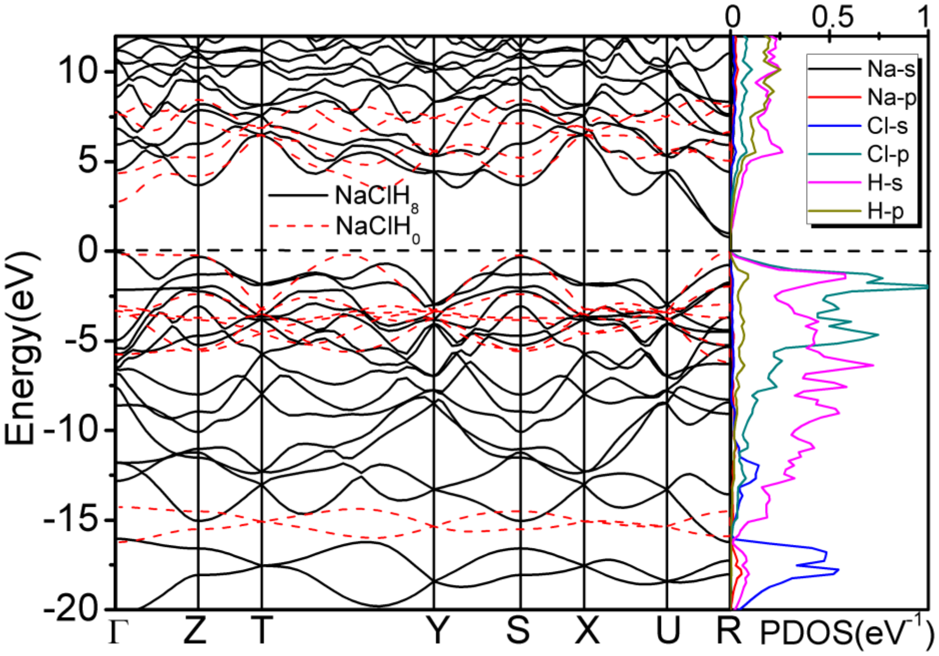

Figure S19. The electronic band structure and PDOS of *Cmmm* phase for $NaCl(H_2)_4$ at 0 GPa. In the left panel, the black solid lines are electronic band structure of $NaClH_2$; the red dashed lines are those of $NaClH_0$ in which all the H_2 molecules are removed from the $NaCl(H_2)_4$ structure. The black line shows the Fermi energy of $NaClH_2$ and $NaClH_0$. The right panel presents the projected DOS of $NaClH_2$.

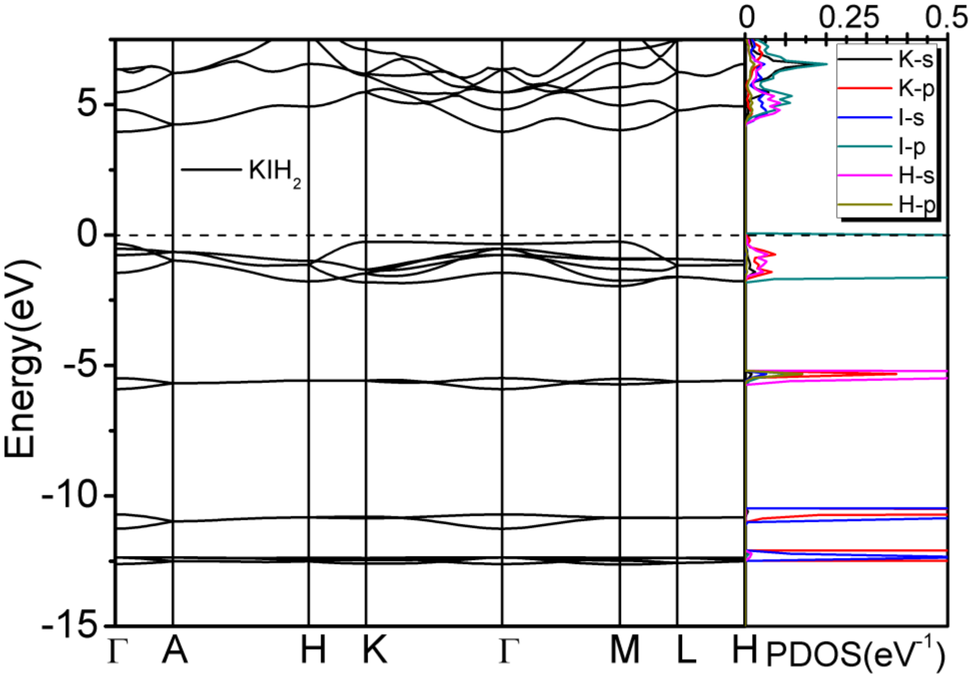

Figure S20. The electronic band structure and PDOS of *P*6$_3$/*mmc* phase for KIH_2 at 0 GPa. In the left panel, the black solid lines are electronic band structure. The black dashed line shows the Fermi energy of KIH_2. The right panel presents the projected DOS of KIH_2.

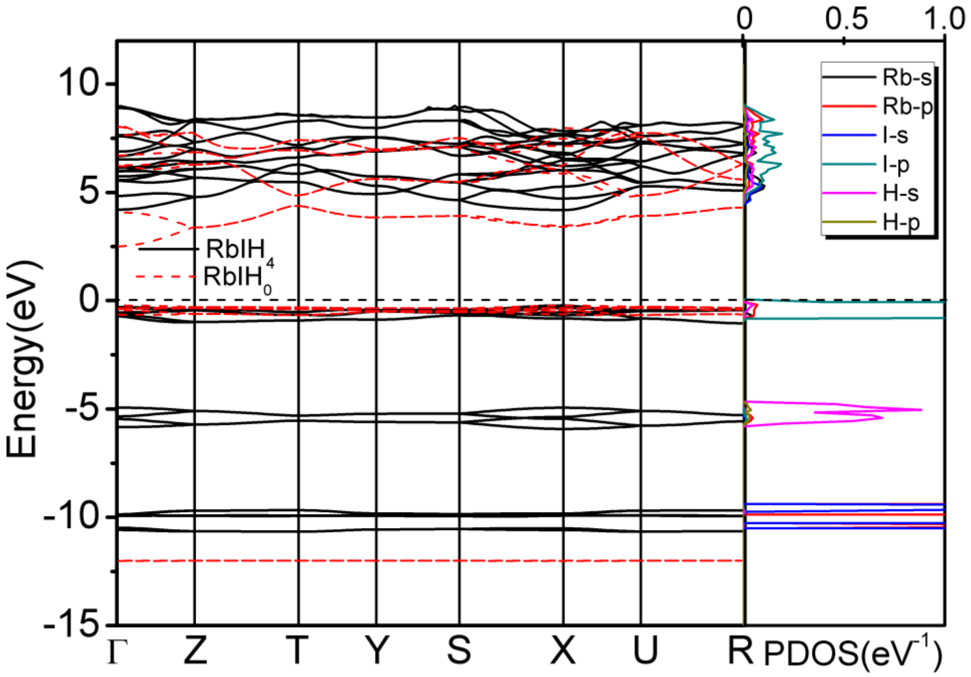

Figure S21. The electronic band structure and PDOS of *Pmmn* phase for $RbI(H_2)_2$ at 0 GPa. In the left panel, the black solid lines are electronic band structure of $RbIH_4$; the red dashed lines are those of $RbIH_0$ in which all the H_2 molecules are removed from the $RbI(H_2)_2$ structure. The black line shows the Fermi energy of $RbI(H_2)_2$ and $RbIH_0$. The right panel presents the projected DOS of $RbIH_4$.

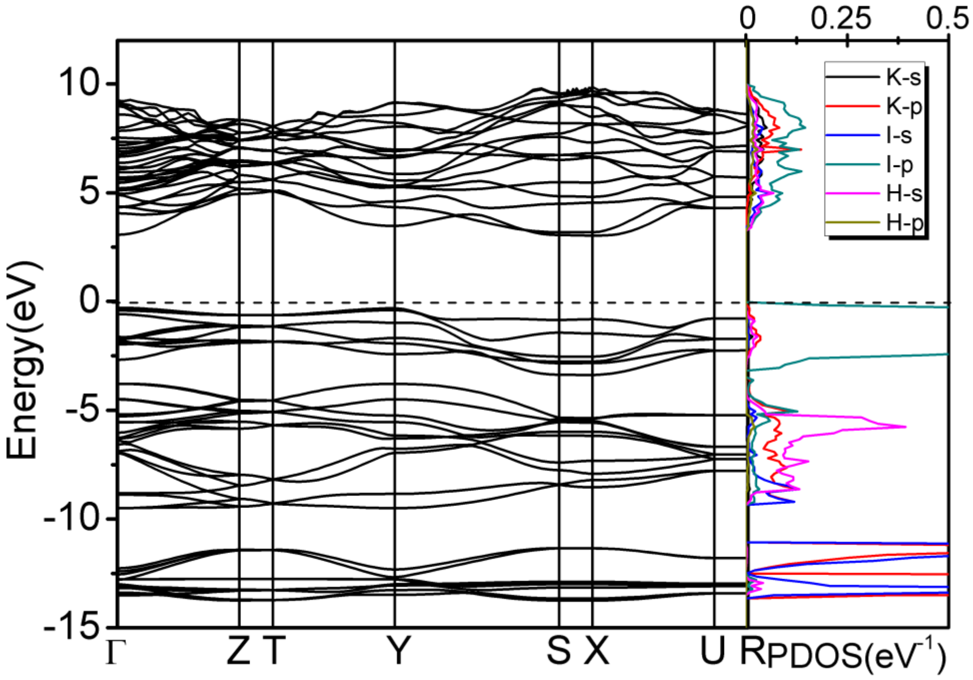

Figure S22. The electronic band structure and PDOS of *Immm* phase for $NaI(H_2)_6$ at 0 GPa. In the left panel, the black solid lines are electronic band structure. The black dashed line shows the Fermi energy of $NaIH_2$. The right panel presents the projected DOS of $NaI(H_2)_6$.

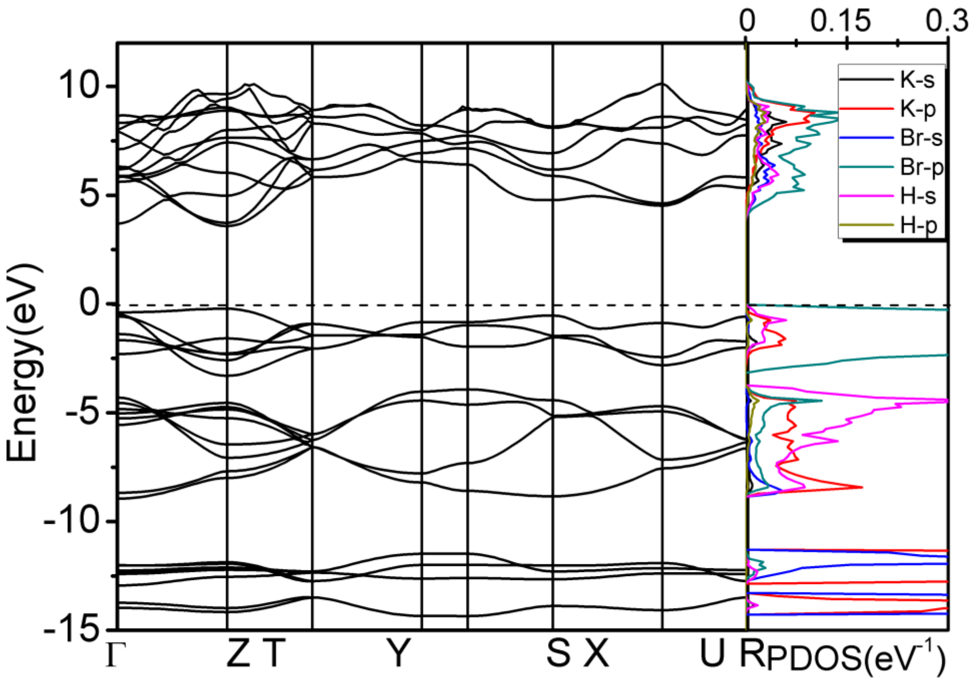

Figure S23. The electronic band structure and PDOS of *Cmmm* phase for $KBr(H_2)_4$ at 0 GPa. In the left panel, the black solid lines are electronic band structure. The black dashed line shows the Fermi energy of $KBr(H_2)_4$. The right panel presents the projected DOS of $KBr(H_2)_4$.

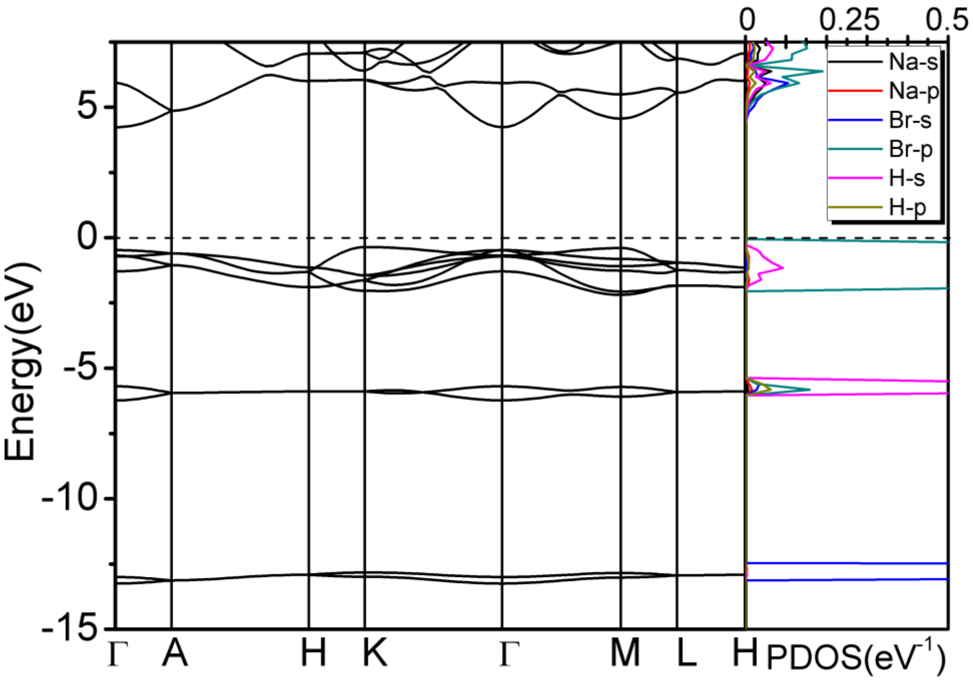

Figure S24. The electronic band structure and PDOS of $P6_3/mmc$ phase for $NaBrH_2$ at 0 GPa. In the left panel, the black solid lines are electronic band structure. The black dashed line shows the Fermi energy of $NaBrH_2$. The right panel presents the projected DOS of $NaBrH_2$.

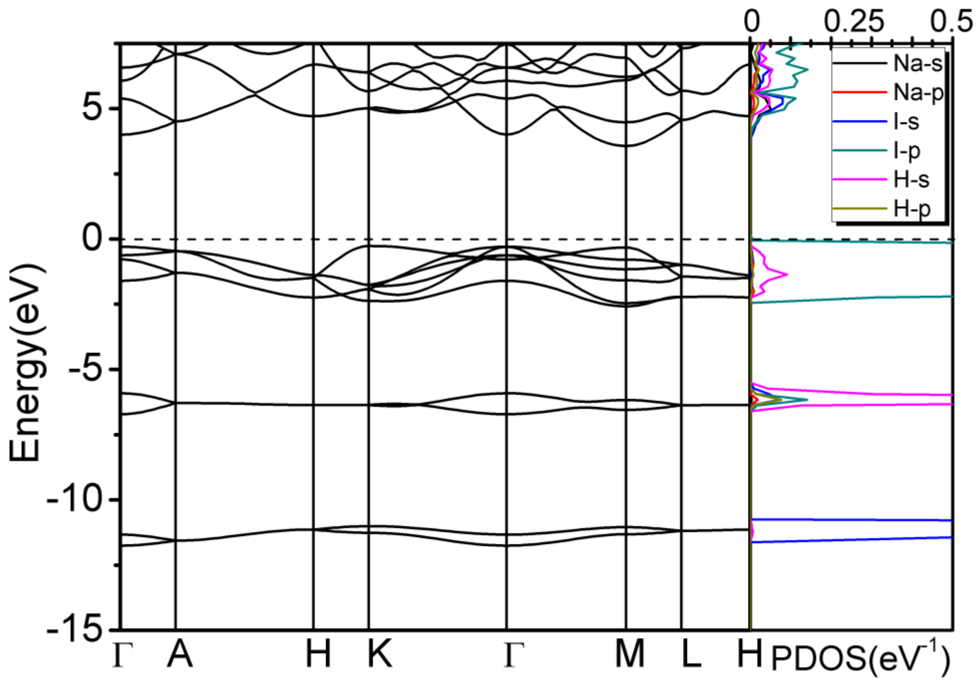

Figure S25. The electronic band structure and PDOS of *I*4₁*md* phase for $NaIH_2$ at 0 GPa. In the left panel, the black solid lines are electronic band structure. The black dashed line shows the Fermi energy of $NaIH_2$. The right panel presents the projected DOS of $NaBrH_2$.

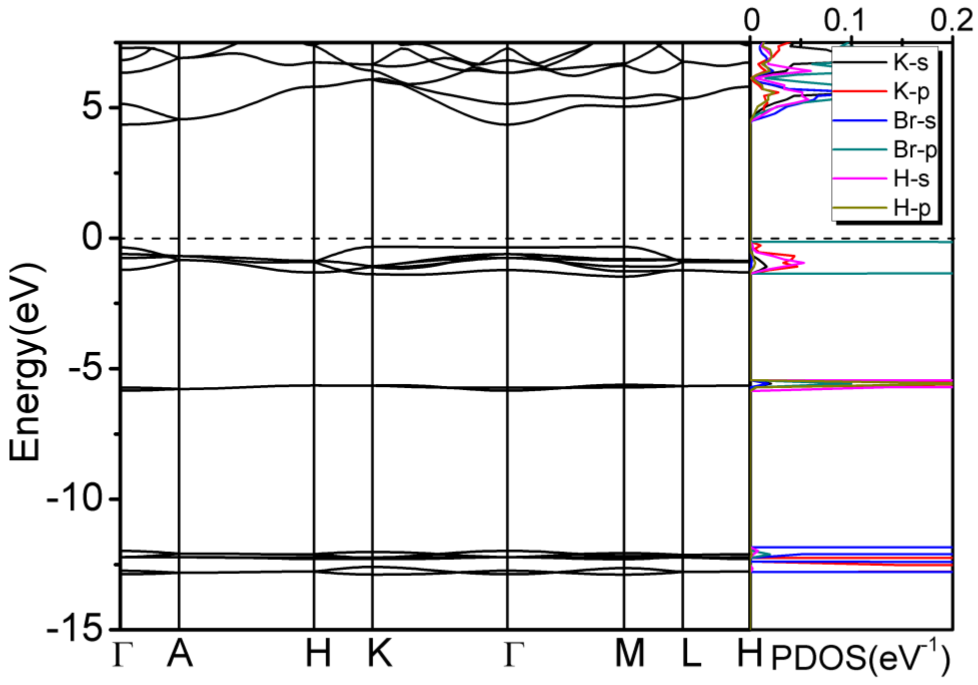

Figure S26. The electronic band structure and PDOS of $P6_3/mmc$ phase for $KBrH_2$ at 0 GPa. In the left panel, the black solid lines are electronic band structure. The black dashed line shows the Fermi energy of $KBrH_2$. The right panel presents the projected DOS of $KBrH_2$.

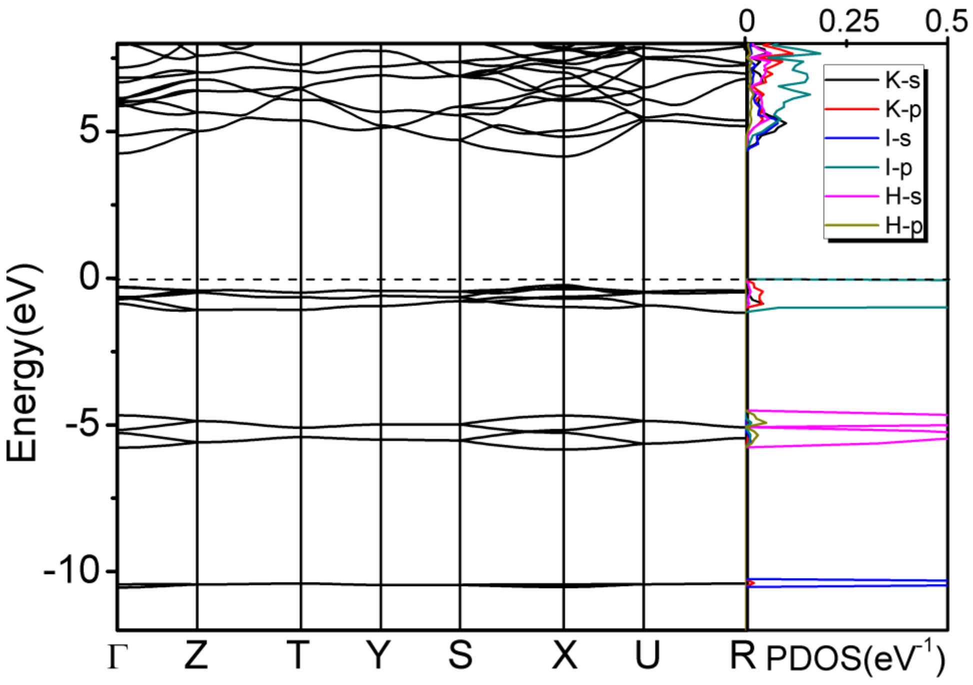

Figure S27. The electronic band structure and PDOS of *Pmmn* phase for KIH_4 at 0 GPa. In the left panel, the black solid lines are electronic band structure of $KI(H_2)_2$; The black line shows the Fermi energy of $KI(H_2)_2$. The right panel presents the projected DOS of $KI(H_2)_2$.

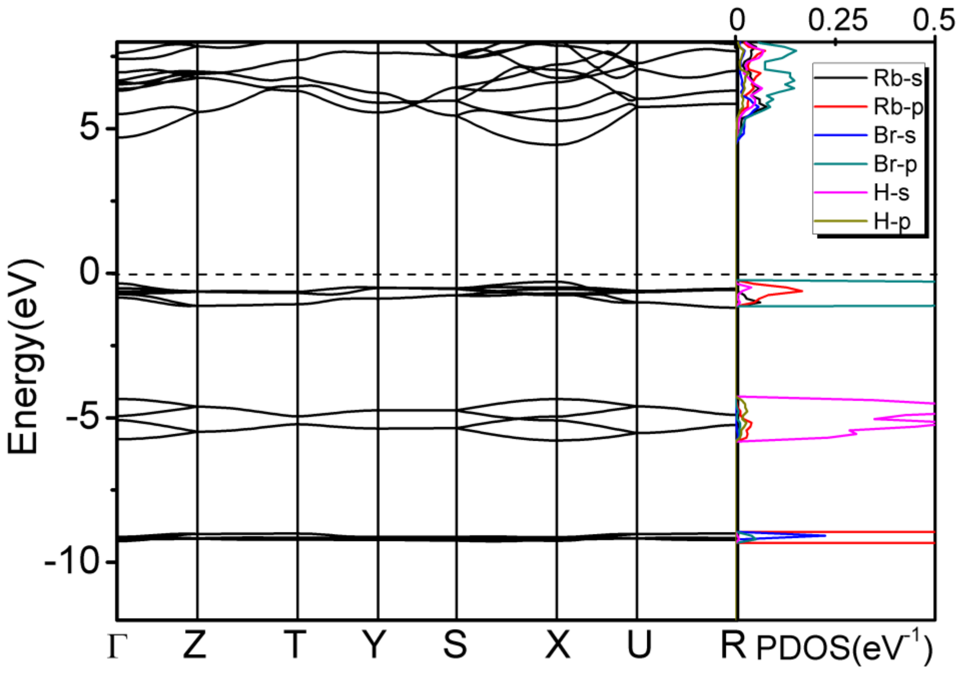

Figure S28. The electronic band structure and PDOS of *Pmmn* phase for $RbBr(H_2)_2$ at 0 GPa. In the left panel, the black solid lines are electronic band structure of $RbBr(H_2)_2$; The black line shows the Fermi energy of $RbBr(H_2)_2$. The right panel presents the projected DOS of $RbBr(H_2)_2$.

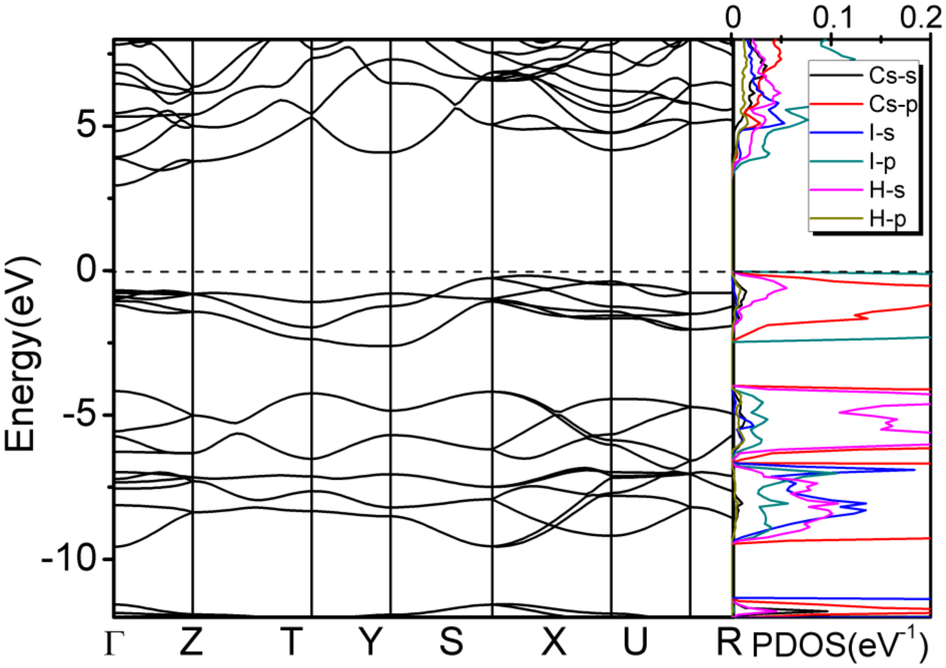

Figure S29. The electronic band structure and PDOS of *Pmmn* phase for $CsI(H_2)_2$ at 20 GPa. In the left panel, the black solid lines are electronic band structure of $CsIH_4$; The black line shows the Fermi energy of $CsI(H_2)_2$. The right panel presents the projected DOS of $CsI(H_2)_2$.

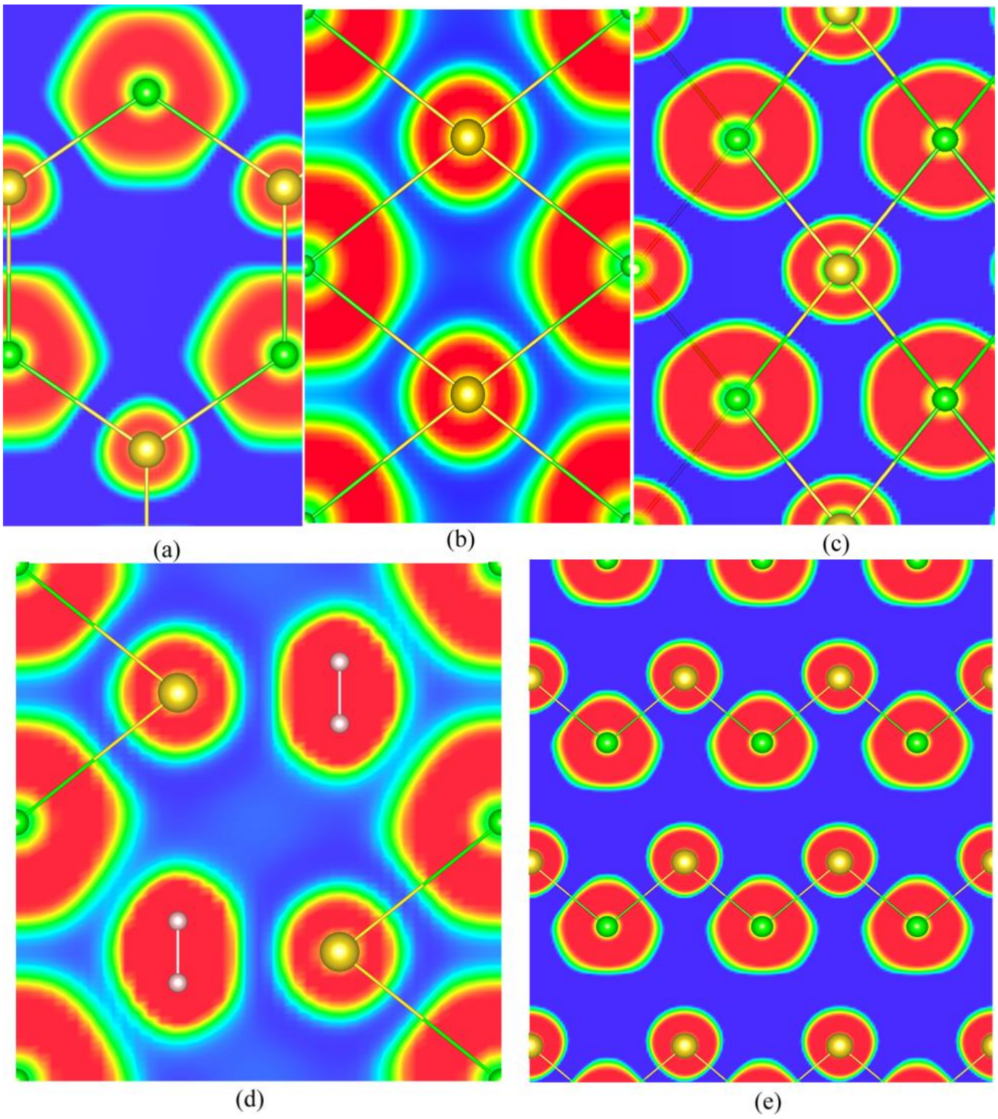

Figure S30. ELF plots for XY-H_2 compounds. (a) Pc phase in (0 0 1) section for $NaClH_2$ at 0 GPa. (b) *Cmmm* phase in (0 0 1) section for $KBr(H_2)_4$ at 20 GPa. (c) *Cmcm* phase in (0 0 1) section for KIH_{10} at 20 GPa. (d) *P*6$_3$/*mmc* phase in (0 0 1) section for KIH_2 at 0 GPa. (e) *Pmmn* phase in (0 0 1) section for $RbI(H_2)_2$ at 0 GPa.

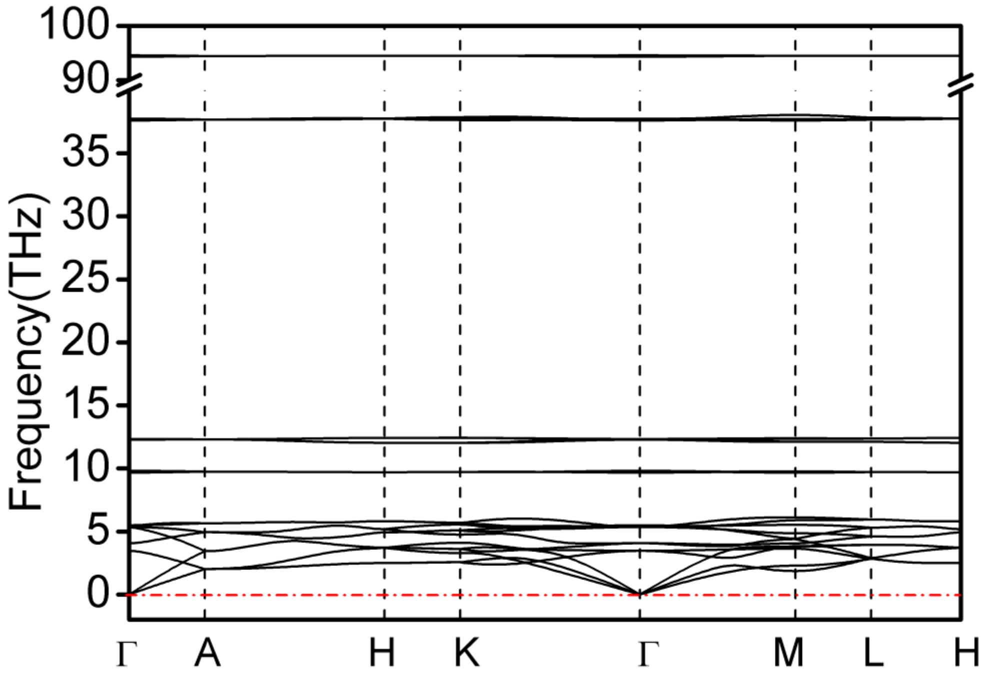

Figure S31. The calculated phonon dispersion curves of stable KIH_2 at 0 GPa. The absence of imaginary frequency of KIH_2 confirms their dynamic stabilities.

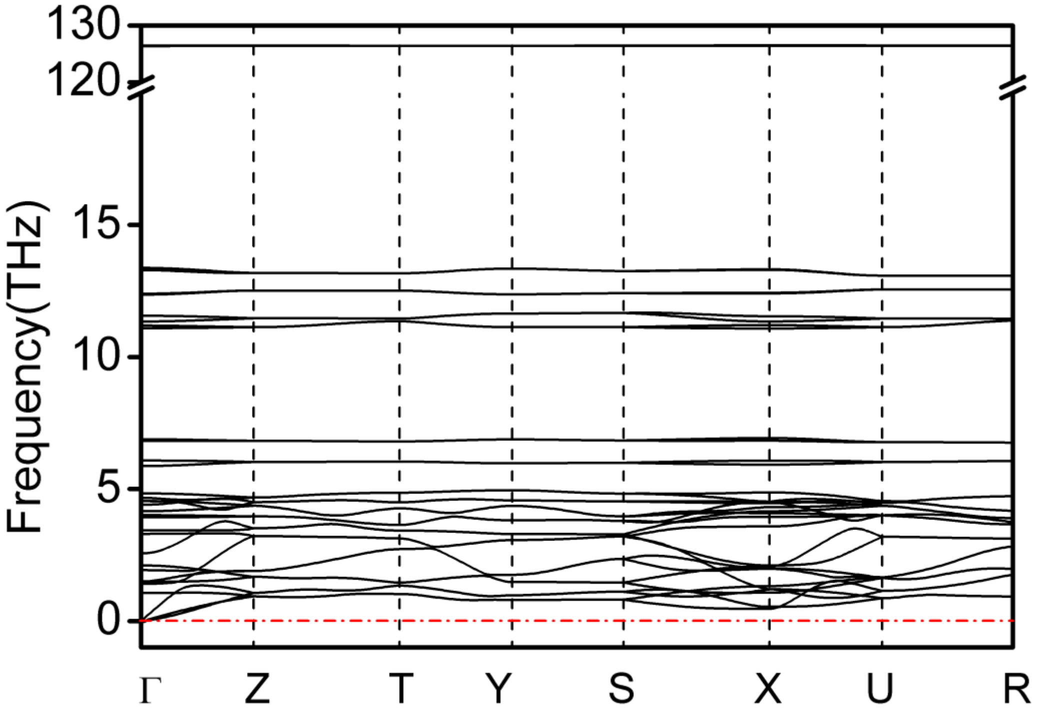

Figure S32. The calculated phonon dispersion curves of stable $KI(H_2)_2$ at 0 GPa. The absence of imaginary frequency of KIH_4 confirms their dynamic stabilities.

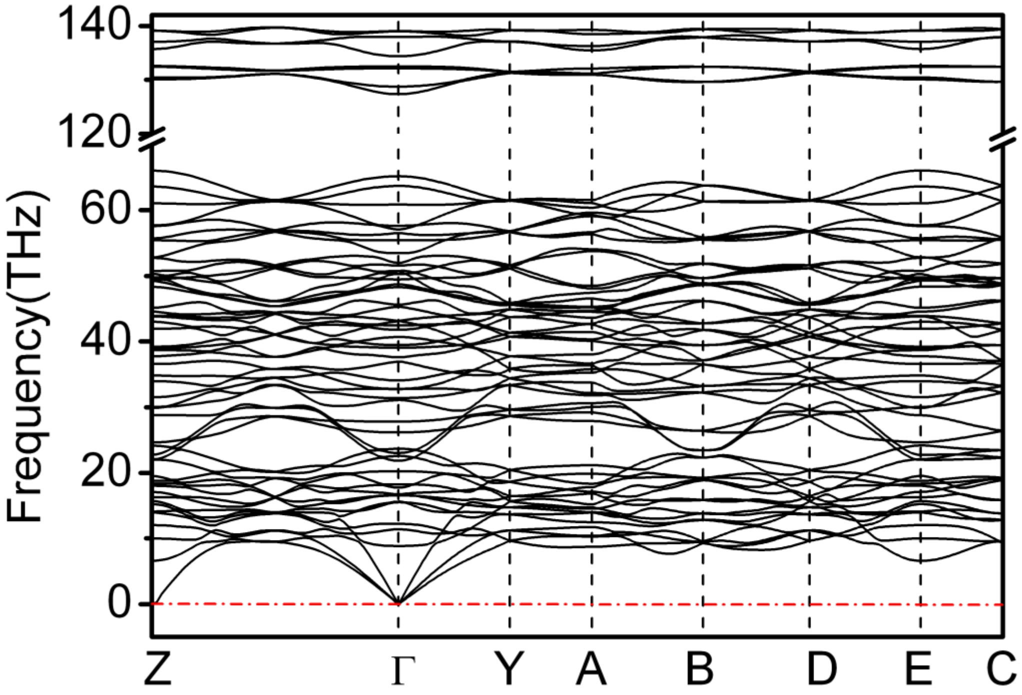

Figure S33. The calculated phonon dispersion curves of stable $NaCl(H_2)_4$ with *Pm* symmetry at 50 GPa. The absence of imaginary frequency of $NaCl(H_2)_4$ confirms their dynamic stabilities.

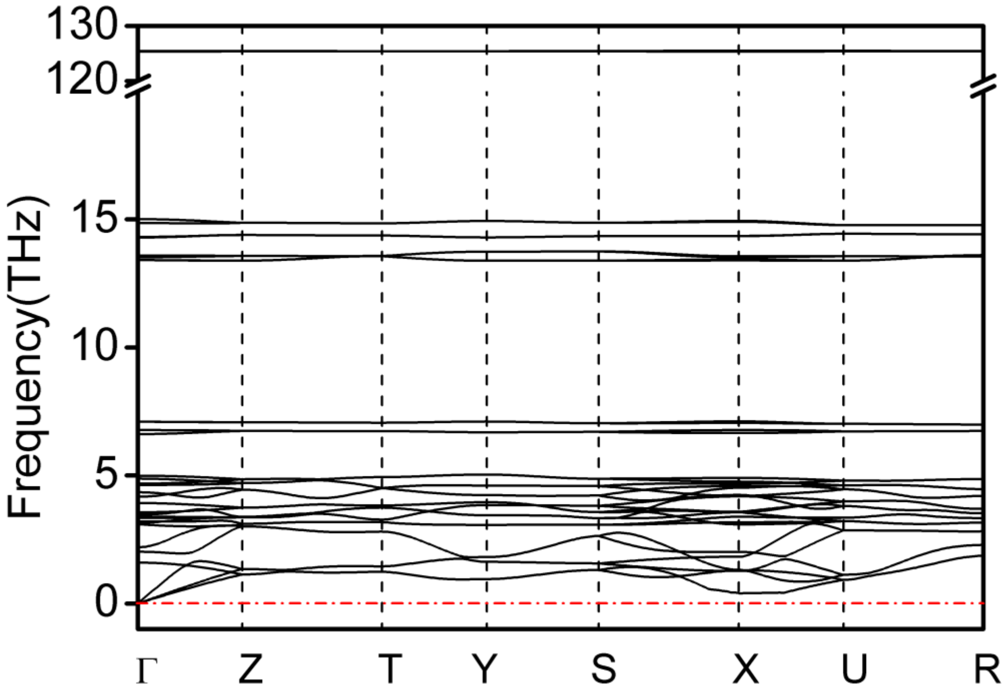

Figure S34. The calculated phonon dispersion curves of stable $RbI(H_2)_2$ at 0 GPa. The absence of imaginary frequency of $RbI(H_2)_2$ confirms their dynamic stabilities.

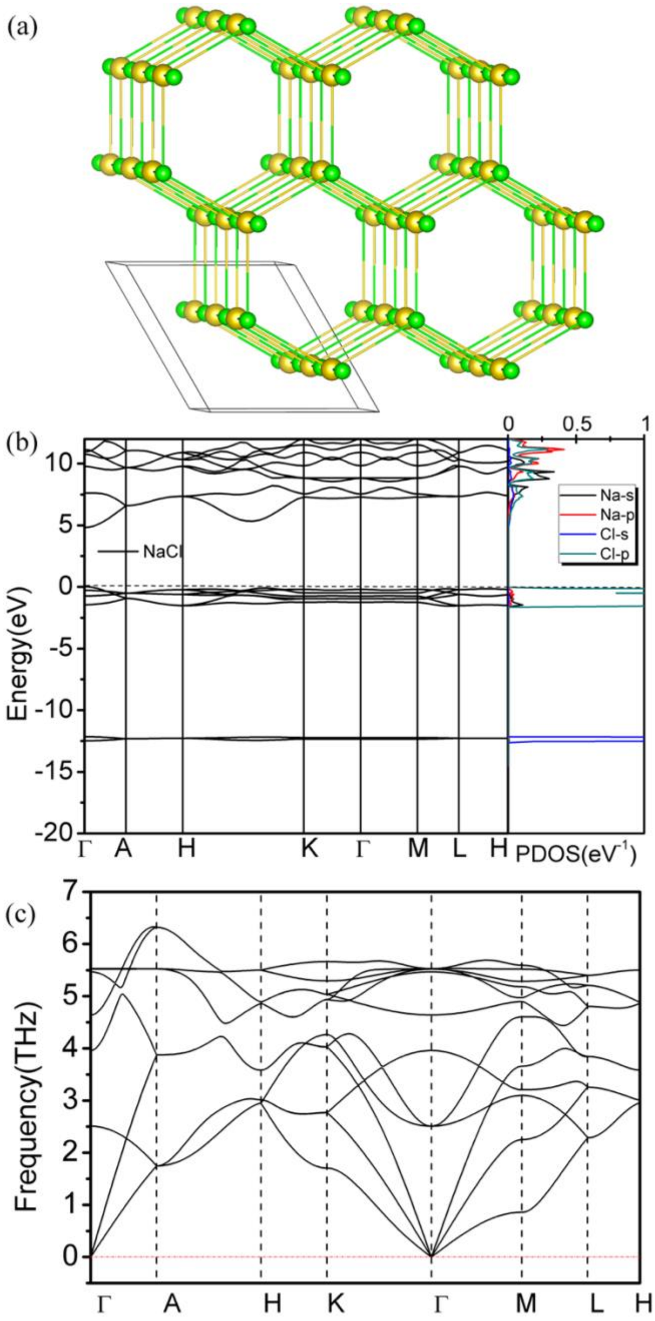

Figure S35. Structure (a), electronic band and PDOS (b), and phonon spectrum (c) of the P63/mmc phase of NaCl at 0 GPa.

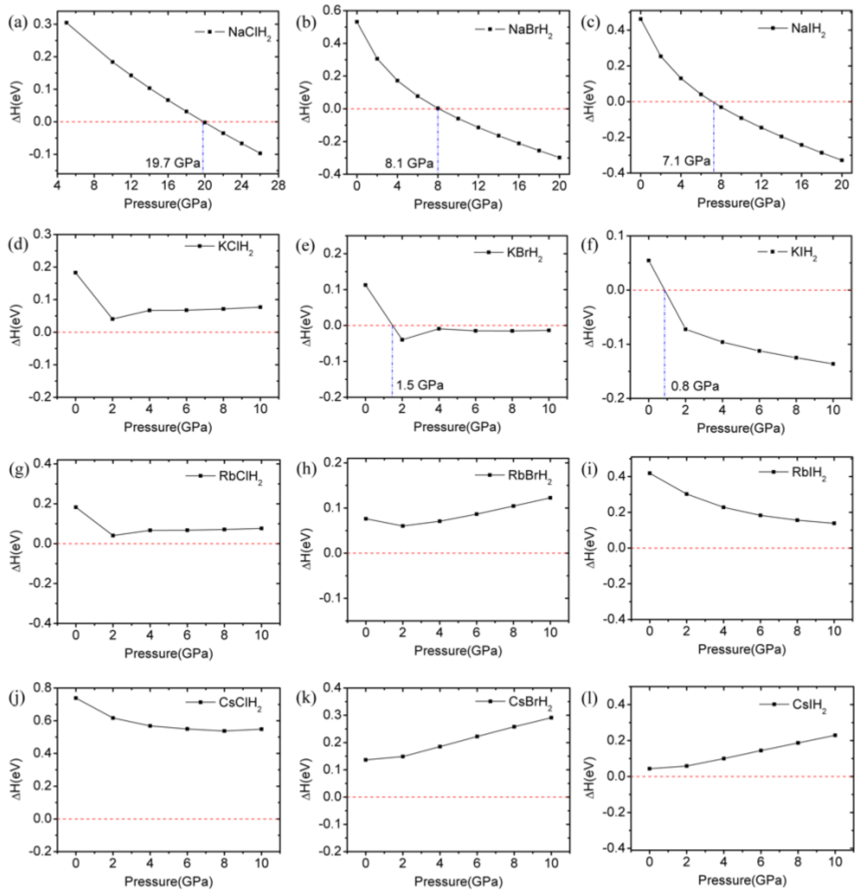

Figure S36. The enthalpy difference of ABH_2 compounds. The black and red dashed lines show the enthalpies of ABH_2 and AB compounds + solid H_2, respectively.

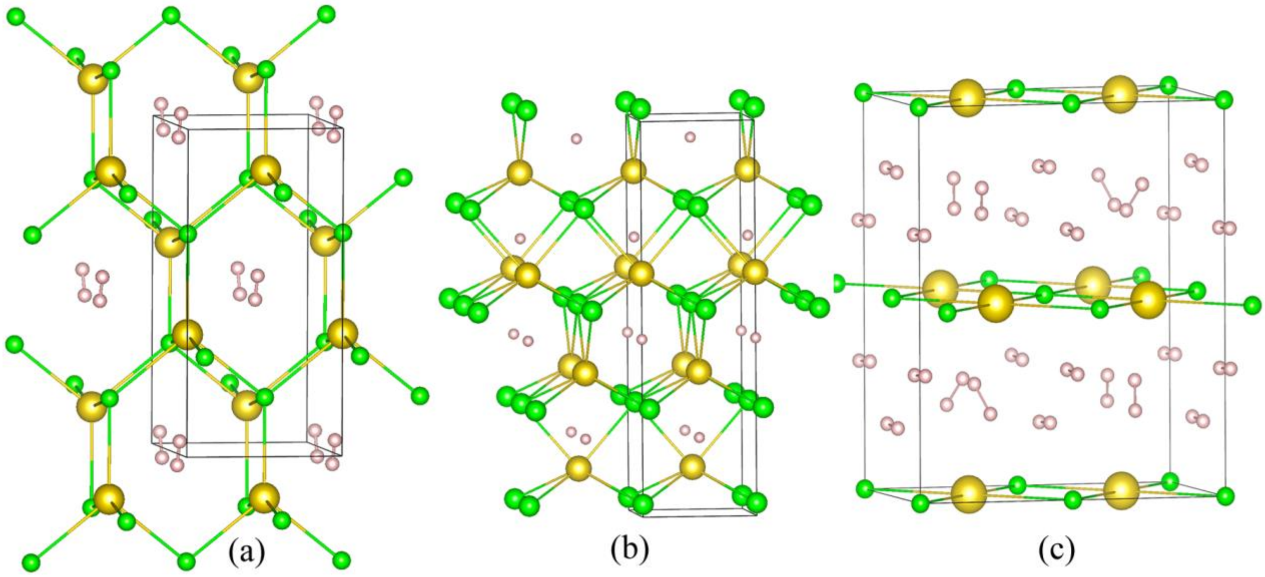

Figure S37. Structures of the *Pc* phase of $NaClH_2$ at 0 GPa (a), $I4_1md$ phase of $NaIH_2$ at 20 GPa (b), and *Cmmm* phase of $NaCl(H_2)_4$ at 80 GPa (c).

Supplemental Table

Table S1 Calculated Bader charges of Na, Cl atoms and H_2 molecule in $NaClH_2$, $NaCl(H_2)_4$ and Na, Cl atoms and N_2 molecule in $NaClN_2$ and Na, Cl atoms and He molecule in $NaClHe_2$.

Phase	Pressure (GPa)	Atoms or molecule	Charge value	δ(e)
***Pc*-NaClH₂**	0	Na	8.12	+0.88
		Cl	7.85	-0.85
		H_2	2.03	+0.03
***P6₃/mmc*-NaClH₂**	50	Na	8.18	+0.82
		Cl	7.76	-0.76
		H_2	2.06	+0.06
***Pm*-NaCl(H₂)₄**	50	Na	8.16	+0.84
		Cl	7.82	-0.81
		H_2	2.03	+0.03
***Cmcm*-NaCl(H₂)₄**	100	Na	8.20	+0.80
		Cl	7.66	-0.66
		H_2	2.04	+0.04
***C2/m*-NaCl(N₂)₅**	100	Na	8.14	+0.86
		Cl	6.72	+0.28
		N_2	10.228	-0.228
***P6₃/mmc*-NaClHe₂**	50	Na	8.17	+0.83
		Cl	7.78	-0.78
		He	2.05	+0.05

δ is the charge transfer from Na to Cl atom.

Table S2. Calculated structural parameters of various NaCl-SM compounds.

	Space group	Lattice Parameters (Å, °)	Atoms	Atomic coordinates (fractional) X	Y	Z
**NaCl-NH₃**	*P-1*	*a*=2.9729	Na1(2i)	0.45126	0.09807	0.80078
**(50 GPa)**		*b*=4.5090	Cl1(2i)	0.78245	0.68899	0.86516
		c=5.4062	N1(2i)	0.90295	0.23533	0.57681
		α=94.9611°	H1(2i)	0.10704	0.42524	0.71895
		β=105.4256°	H3(2i)	0.35332	0.69097	0.53709
		γ=107.9347°	H4(2i)	0.11644	0.22332	0.46225
**NaCl-CO₂**	*Cm*	*a*=6.9097	Na1(4b)	0.88460	0.30611	0.21618
**(200 GPa)**		*b*=4.4917	O3(4b)	0.13847	0.75107	0.47667
		c=3.9517	Cl1(2a)	0.59216	0.50000	0.00373
		α=*γ*=90.0000°	C2(2a)	0.55019	0.50000	0.60887
		β=87.4171°	O2(2a)	0.89569	0.50000	0.72062
			Cl2(2a)	0.69428	-0.00000	0.00666
			C1(2a)	0.57424	-0.00000	0.65017
			O1(2a)	0.88053	-0.00000	0.54923

**$(NaCl)_2$-H_2O**	*C2/c*	*a* = 6.76910	Na1(8f)	0.881810	0.620370	0.995860
**(200 GPa)**		*b* = 3.90920	Cl1(8f)	0.541550	0.379440	0.646850
		c = 9.46000	H1(8f)	0.325390	0.058670	0.685190
		α= *γ*=90.0000°	O1(4e)	0.500000	0.895960	0.750000
		β= 147.6514°				
**NaCl-H_2**	*Pc*	*a*=7.3829	Na1(2a)	-0.02167	0.35174	0.38338
**(0 GPa)**		*b*=9.0376	Na3(2a)	-0.52219	0.85178	-0.11691
		c=5.6347	Cl1(2a)	-0.52178	0.82885	0.38374
		α=*γ*=90.0000°	Cl3(2a)	-0.02193	0.32896	0.88352
		β=139.9604°	H1(2a)	-0.01941	0.03271	0.18067
			H2(2a)	-0.51992	0.46339	0.16628
			H5(2a)	-0.52348	0.46482	-0.40434
			H7(2a)	-0.02391	0.96061	0.11000
**NaCl-H_2**	*$P6_3mmc$*	*a*=*b*=3.6291	Na1(2c)	0.666667	0.333333	0.750000
**(20 GPa)**		*c*=6.6315	Cl1(2a)	0.000000	0.000000	0.000000
		α=*β*=90.0000°	H1(2d)	0.666667	0.333333	0.250000
		γ=120.0000°	H2(0)	0.333333	0.666667	0.750000
**NaBr-$(H_2)_2$**	*Pmmn*	*a=3.0772*	Na1(2b)	0.00000	0.50000	-0.29206
**(30 GPa)**		*b=4.4871*	Br1(2a)	0.00000	0.00000	-0.11535
		c=4.2372	H1(4e)	0.50000	0.67435	-0.45225
		α=β=γ=90.0000°	H5(4e)	0.00000	0.73172	-0.69120
**NaCl-$(H_2)_4$**	*Pm*	*a*=4.1550	Na1(1b)	0.267670	0.500000	0.663110
**(50 GPa)**		*b=3.1460*	Cl2(1b)	-0.066140	0.500000	0.006510
		c=4.1970	H3(1b)	0.670730	0.500000	0.450160
		α=*γ*=90.0000°	H4(1b)	0.530230	0.500000	0.258980
		β=118.9100°	H5(1a)	-0.084520	-0.000000	0.406440
			H6(1a)	-0.076920	-0.000000	0.588050
			H7(1a)	0.532260	-0.000000	0.021060
			H8(1a)	0.527720	-0.000000	0.585980
			H9(1a)	0.350070	-0.000000	0.017300
			H10(1a)	0.350070	-0.000000	0.408580
**NaI-$(H_2)_6$**	*Immm*	*a*=9.4407	H1(8n)	-0.33628	-0.31549	-0.00000
**(20 GPa)**		*b*=4.6455	H3(8n)	-0.12343	-0.41948	0.00000
		c=3.5829	H7(8n)	-0.28511	0.19295	-0.00000
		α=*β*=*γ*=90.0000°	Na1(2b)	-0.00000	-0.50000	-0.50000
			I1(2a)	0.00000	0.00000	0.00000
**NaCl-$(N_2)_5$**	*C2/m*	*a*=8.1816	N1(8j)	0.29102	-0.32796	0.35078
**(100 GPa)**		*b*=6.2393	N5(8j)	1.33337	-0.89504	1.97267
		c=5.5796	Na1(2d)	0.50000	0.00000	0.50000
		α=*γ*=90.0000°	Cl1(2d)	0.00000	0.00000	0.00000

	β=139.5728°	N7(4i)	1.14011	-0.00000	1.45849

References

1. Wang, Y., Lv, J., Zhu, L. & Ma, Y. Crystal structure prediction via particle-swarm optimization. *Phys. Rev. B* **82,** 094116 (2010).
2. Wang, Y., Lv, J., Zhu, L. & Ma, Y. CALYPSO: A method for crystal structure prediction. *Comput. Phys. Commun.* **183,** 2063–2070 (2012).
3. Lv, J., Wang, Y., Zhu, L. & Ma, Y. Predicted Novel High-Pressure Phases of Lithium. *Phys. Rev. Lett.* **106,** 015503 (2011).
4. Zhu, L. *et al.* Substitutional Alloy of Bi and Te at High Pressure. *Phys. Rev. Lett.* **106,** 145501 (2011).
5. Wang, Y. *et al.* High pressure partially ionic phase of water ice. *Nat. Commun.* **2,** 563–5 (2011).
6. Wang, H., John, S. T., Tanaka, K., Iitaka, T. & Ma, Y. Superconductive sodalite-like clathrate calcium hydride at high pressures. *Proc. Natl. Acad. Sci. USA* **109,** 6463–6466 (2012).
7. Wang, X. *et al.* Cagelike Diamondoid Nitrogen at High Pressures. *Phys. Rev. Lett.* **109,** 175502 (2012).
8. Zhu, L., Liu, H., Pickard, C. J., Zou, G. & Ma, Y. Reactions of xenon with iron and nickel are predicted in the Earth's inner core. *Nat. Chem.* **6,** 644–648 (2014).
9. Guillaume, C. L. *et al.* Cold melting and solid structures of dense lithium. *Nat. Phys.* **7,** 211–214 (2011).
10. Pickard, C. J. & Needs, R. J. Ab initio random structure searching. *J. Phys.: Condens. Matter* **23,** 053201 (2011).
11. Kresse, G. Software VASP, Vienna, 1999; G. Kresse, J. Furthmüller. *Phys. Rev. B* **54,** (1996).
12. Clark, S. J. *et al.* First principles methods using CASTEP. *Zeitschrift für Kristallographie-Crystalline Materials* **220,** 567–570 (2005).
13. Blöchl, P. E. Projector augmented-wave method. *Phys. Rev. B* **50,** 17953 (1994).
14. Perdew, J. P., Burke, K. & Ernzerhof, M. Generalized gradient approximation made simple. *Phys. Rev. Lett.* **77,** 3865 (1996).
15. Perdew, J. P. & Wang, Y. Accurate and simple analytic representation of the electron-gas correlation energy. *Phys. Rev. B* **45,** 13244 (1992).
16. Becke, A. D. & Edgecombe, K. E. A simple measure of electron localization in atomic and molecular systems. *J. Chem. Phys.* **92,** 5397 (1990).
17. Parlinski, K., Li, Z. Q. & Kawazoe, Y. First-principles determination of the soft mode in cubic ZrO_2. *Phys. Rev. Lett.* **78,** 4063 (1997).
18. Togo, A., Oba, F. & Tanaka, I. First-principles calculations of the ferroelastic transition between rutile-type and $CaCl_2$-type SiO_2 at high pressures. *Phys. Rev. B* **78,** 134106 (2008).
